# Motor neuron intrinsic and extrinsic mechanisms contribute to the pathogenesis of *FUS*-associated amyotrophic lateral sclerosis

**DOI:** 10.1007/s00401-017-1687-9

**Published:** 2017-02-28

**Authors:** Jelena Scekic-Zahirovic, Hajer El Oussini, Sina Mersmann, Kevin Drenner, Marina Wagner, Ying Sun, Kira Allmeroth, Stéphane Dieterlé, Jérôme Sinniger, Sylvie Dirrig-Grosch, Frédérique René, Dorothee Dormann, Christian Haass, Albert C. Ludolph, Clotilde Lagier-Tourenne, Erik Storkebaum, Luc Dupuis

**Affiliations:** 1 Inserm, UMR-S1118, 67085 Strasbourg, France; 2 Faculté de Médecine, UMR-S1118, Université de Strasbourg, 67085 Strasbourg, France; 3 Molecular Neurogenetics Laboratory, Max Planck Institute for Molecular Biomedicine, Muenster, Germany; 4 Faculty of Medicine, University of Muenster, Muenster, Germany; 5 Department of Neurosciences, Ludwig Institute for Cancer Research, University of California, San Diego, USA; 6 BioMedical Center (BMC), Cell Biology, Ludwig-Maximilians-Universität München, Munich, Germany; 7 Munich Cluster for Systems Neurology (SyNergy), Munich, Germany; 8 BioMedical Center (BMC), Biochemistry, Ludwig-Maximilians-Universität München, Munich, Germany; 9 German Center for Neurodegenerative Diseases (DZNE) Munich, Munich, Germany; 10 Department of Neurology, Ulm University, Ulm, Germany; 11 Department of Neurology, Harvard Medical School, Massachusetts General Hospital, Charlestown, MA 02129 USA; 12 Broad Institute of Harvard University and MIT, Cambridge, MA 02142 USA

**Keywords:** Amyotrophic lateral sclerosis, Fronto-temporal dementia, Mouse models, Non-cell autonomous mechanisms, RNA-binding proteins

## Abstract

Motor neuron-extrinsic mechanisms have been shown to participate in the pathogenesis of ALS-SOD1, one familial form of amyotrophic lateral sclerosis (ALS). It remains unclear whether such mechanisms contribute to other familial forms, such as TDP-43 and FUS-associated ALS. Here, we characterize a single-copy mouse model of ALS-*FUS* that conditionally expresses a disease-relevant truncating FUS mutant from the endogenous murine *Fus* gene. We show that these mice, but not mice heterozygous for a *Fus* null allele, develop similar pathology as ALS-*FUS* patients and a mild motor neuron phenotype. Most importantly, CRE-mediated rescue of the *Fus* mutation within motor neurons prevented degeneration of motor neuron cell bodies, but only delayed appearance of motor symptoms. Indeed, we observed downregulation of multiple myelin-related genes, and increased numbers of oligodendrocytes in the spinal cord supporting their contribution to behavioral deficits. In all, we show that mutant FUS triggers toxic events in both motor neurons and neighboring cells to elicit motor neuron disease.

**Electronic supplementary material:**

The online version of this article (doi:10.1007/s00401-017-1687-9) contains supplementary material, which is available to authorized users.

## Introduction

Amyotrophic lateral sclerosis (ALS) is an incurable neurodegenerative disease clinically characterized by a preferential loss of upper and lower motor neurons, resulting in progressive weakness of skeletal muscles, atrophy, paralysis and death due to respiratory failure [42]. Recent advances in human genetics identified mutations in almost 40 genes associated with ALS, and these familial cases together account for about 10% of ALS cases [62, 79].

Heterozygous mutations in the *FUS (Fused in sarcoma)* gene, encoding the RNA-binding protein FUS, are the major cause of juvenile forms of ALS [14, 34, 45, 84]. In ALS-*FUS* patients, the FUS protein accumulates in the cytoplasm in a dimethylated form [19, 75]. FUS is functionally related to TDP-43 (TAR DNA-binding protein 43), the major protein found in ubiquitin-positive inclusions of ALS patients [59], and, like TDP-43, FUS is a nuclear protein involved in multiple steps of gene expression, including mRNA transcription, splicing, transport and translation [49, 56]. In neurons, FUS is found in axons [69], dendrites and at excitatory synapses [24] as well as in RNA transporting granules [4, 10]. Several recent studies demonstrated that the complete loss of FUS protein, either in adult mice or perinatally, was not sufficient to trigger motor neuron degeneration [44, 68, 72, 82]. Contrasting with this, overexpression of FUS, either wild type or mutant, is able to trigger motor neuron degeneration, suggesting that the mutant protein gains a toxic function leading to aggressive neurodegeneration [55, 64, 71–73]. Importantly, the majority of *FUS* mutations are missense changes clustered in the C-terminal nuclear localization sequence (NLS) or frameshift and stop mutations that truncate the NLS [16]. This impairs the binding of FUS to the nuclear import receptor Transportin, and thus interferes with import of FUS in the nucleus, resulting in the cytoplasmic accumulation of FUS [20]. Consistent with a critical role of nuclear import of FUS, the mutations leading to the most severe forms of ALS are truncating or frameshift mutations in *FUS* causing the complete deletion of the NLS [3, 11, 16, 87, 88, 96]. These aggressive *FUS* mutations lead to extensive FUS redistribution to the cytoplasm and age of onset was correlated with the degree of cytosolic mislocalization of FUS [20]. Together, these findings strongly suggest that neurodegeneration is directly related to the altered subcellular localization of FUS.

To study the mechanisms of ALS-*FUS* in a physiologically relevant manner, we recently generated a conditional knock-in mouse model (*Fus* ^*ΔNLS*^ mice) in which the NLS of FUS is deleted [68]. We have shown that FUS is completely mislocalized to the cytoplasm in mice homozygous for the *Fus* ^*ΔNLS*^ mutation [68], leading to motor neuron degeneration in neonates. However, homozygous knock-in mice were lethal at birth, thus precluding the analysis of aging mice homozygous for the *Fus* mutation. Here, we studied heterozygous *Fus* ^*ΔNLS/*+^ mice, as a mouse model carrying a genetic defect that mimics the genetic situation in human ALS-*FUS* patients. Analysis of these mutant mice revealed progressive motor neuron degeneration and neuropathological changes that faithfully model several key aspects of ALS-*FUS*, including ubiquitin pathology and cytoplasmic accumulation of dimethylated FUS. Motor neuron death appeared cell autonomous, yet the motor phenotype of these mice was only delayed when the mutation was rescued solely within motor neurons, and axonal defects were still present. Further, we identified alterations in genes involved in myelin structure and function, and showed altered abundance of oligodendrocytes in the spinal cord supporting the contribution of these cells to the disruption of axonal integrity and the motor phenotype. Thus, while expression of mutant FUS within motor neurons is necessary for cell death, motor symptoms are caused by the concerted action of mutant FUS in motor neurons and other cell types, including oligodendrocytes.

## Materials and methods

### Animal housing and genotyping

Wild type and heterozygous *Fus* ^∆NLS/+^ mice and heterozygous *Chat*-CRE mice, generated as described previously [68], were bred and housed in the central animal facility of the Faculty of Medicine of Strasbourg, with a regular 12-h light and dark cycle (light on at 7:00 am) under constant conditions (21 ± 1 °C; 60% humidity). Standard laboratory rodent food and water were available ad libitum throughout all experiments. Wild type and heterozygous *Fus* ^+*/*−^ mice, generated as described previously [68], were bred and housed in the animal facility of the Max Planck Institute for Molecular Biomedicine, with a regular 12-h light and dark cycle. Mice were genotyped by PCR of genomic DNA from tail biopsies as described previously [68].

10- to 22-month-old male littermates of each genotype (*Fus* ^+*/*+^, *Fus* ^*ΔNLS/*+^ and *Fus* ^+*/*−^) on a pure genetic background (C57/Bl6) were subjected to behavioral tests and molecular analyses. Behavioral test were done during the light phase of their light/dark cycle.

### Compliance with ethical standards

These protocols were approved by the local ethical committees (Cremeas in Strasbourg, LANUV NRW in Muenster), under reference number AL/27/34/02/13; 84-02.04.2011.A100 and 84-02.04.2016.A166.

### Subcellular fractionation and western blotting

Nuclear and cytoplasmic fractions were prepared from fresh spinal cord tissue using the NE-PER^®^ Nuclear and Cytoplasmic Extraction reagents (Thermo Scientific) according to the manufacturer. Protein concentration was quantitated using the BCA protein assay kit (Pierce). Equal amounts of protein (10 µg for nuclear and 30 µg for cytoplasmic fraction) were loaded in each well of a gradient 4–20% SDS-PAGE gel, separated and transferred onto a 0.45 µm nitrocellulose membrane (BioRad) using a semi-dry Transblot Turbo transfer system (BioRad). Membranes were saturated with 10% non-fat milk in PBS and were then probed with the following primary antibodies: goat anti-FUS against the N-terminal part of protein (ProteinTech 11570; 1:1000), rabbit anti-FUS against the C-terminal part of protein (Bethyl A300-294A, 1:10000) and rat anti-di-methylated FUS (ADMA, 1:1000) [19, 75] all diluted in 3% non-fat milk in PBS. Blots were incubated with horseradish peroxidase (HRP)-labeled secondary antibodies anti-goat (Sigma A5420), anti-rabbit (P.A.R.I.S. BI2407), anti-sheep (Chemicon AP147) and anti-rat (Rockland 612-1102), all secondary antibodies were diluted 1:5000 in PBS. Antibodies rabbit anti-HDAC1 (Bethyl A300-713A, 1:1000) was used as loading control for nuclear fraction and mouse anti sheep SOD1 (Merk 574597, 1:1000) was used as loading control for cytoplasmic fraction. Blots were analyzed with chemiluminescence (ECL; Luminata Forte Kit, Millipore WBLUF0500) using the Molecular Imager Chemidoc XRS (Biorad) as detection system and total protein as loading controls.

For western blot on protein extracts from spinal cord of *Fus* ^+*/*−^ and *Fus* ^+*/*+^ mice, rabbit anti-FUS (Bethyl A300-294A) was used as a primary antibody in a 1:800 dilution. A mouse monoclonal anti-beta-tubulin antibody (clone E7, DSHB, 1:2000) was used as a loading control. As secondary antibodies, HRP-conjugated anti-rabbit (Promega W4011) and anti-mouse (Promega W4021) antibodies were used in a 1:2500 dilution.

### Spinal cord histology

Animals were anesthetized with ketamine (Imalgene 80 mg/kg; Merial, Lyon, France) and xylazine (Rompun 20 mg/kg; Bayer, Lyon, France) and perfused transcardially with 4% paraformaldehyde (PFA) in 0.1 M phosphate buffer (PB), pH 7.4. Spinal cord were dissected and fixed by immersion in 4% paraformaldehyde in 0.1 M phosphate buffer pH 7.4 overnight. The lumbar part of spinal cords (L1–L5) was cryoprotected in 30% sucrose, snap frozen in melting isopentane, and embedded in TissueTek (O.C.T.Compound, SAKURA#4583). Cryosections (Leica CM 3050S) of 16 µm were obtained for histological analysis (10 sections per animal).

Spinal cord sections were stained using rabbit anti-FUS antibody against the FUS N-terminal part (ProteinTech 11570, 1:100), goat anti-ChAT (Millipore AB144-P, diluted 1:50), and Hoechst (Sigma 33342, 1:1000) followed by fluorescently labeled secondary antibodies donkey anti-rabbit Alexa 488 (Jackson A21206), goat anti-rabbit Alexa 488 (Invitrogen A11008), goat anti-mouse Alexa 594 (Invitrogen A11005) and donkey anti-goat Alexa 594 (Molecular Probes A11058) diluted 1:500.

Other antibodies used for spinal cord staining included rat anti-di-methylated FUS (ADMA, 1:100), rabbit anti UBIQUITIN (Abcam ab179434, 1:100), mouse anti-ubiquitin (Millipore MAB1510, 1:100), guinea pig anti-P62 (Progen GP-62C, 1:100), rabbit anti-FUS (Bethyl A300-302A, 1:150), mouse anti-NeuN (clone A60, Millipore MAB377, 1:500), mouse anti-CNPase (Sigma C-5922, 1:100) and rabbit anti-carbonic anhydrase II (kind gift of Dr S. Ghandour, 1:200 for IF and 1:1000 for DAB staining) [13, 35, 74].

### Imaging

Single-layer images were acquired using a laser-scanning microscope (confocal Leica SP5 Leica Microsystems CMS GmbH) equipped with ×63 oil objective (NA1.4). Excitation rays are sequential: Hoechst 33342 was excited using diode 405 nm, Alexa 488 by the argon laser 488 nm, Alexa 594 by diode 561 nm and Alexa 647 by the Helium Neon laser 633 nm. Emission bandwidths were 410–470 nm for Hoechst 33342, 520–550 nm for Alexa 488, 570–620 nm for Alexa 594, and 650–690 nm for Alexa 647. Intensity of FUS fluorescent staining was measured using the software Nis Elements version 4.0.

### Motor coordination and muscle strength analysis

Mice were followed weekly for general health, neurological symptoms, body weight, grip test and accelerating rotarod performances starting from weaning (4 weeks of age) until 22 months of age as described previously [39]. Briefly, mouse motor performance was assessed using rotarod (Ugobasile model 7650). Each session consisted of three tests of 300 s with an acceleration period (4–20 rpm during 150 s) followed by 150 s at constant speed. To evaluate muscle strength, we used a grip strength meter test (Bioseb, ALG01; France). The muscle force (in Newton) was measured three times per mouse. Results are presented as one measurement point per month.

### Inverted grid test

The four limbs hang test uses a wire grid system to non-invasively measure the ability of mice to use sustained limb tension to oppose their gravitational force. The procedure measures 4 limbs hang time in seconds as well as the minimal holding impulse. Each mouse was placed at the simple cage grid and was allowed to accommodate to this environment for 3–5 s before the grid was inverted and held approximately 35 cm over a mouse cage containing 5–6 cm of bedding (wood chips). Each of these holding periods began with all four paws of the mouse grasping the grid. The wire grid hanging time (or “hang time”) was defined as the amount of time that it takes the mouse to fall down from the inverted grid and was measured visually with a stop watch. In each session, the procedure was repeated three times with approximately 10 min between each assessment of holding time. The mouse body weight was obtained shortly before the test. The physical impulse (holding impulse) is the hanging time multiplied by the gravitational force of the mouse [body mass (g) × 0.00980665 N/g × hanging time (s)]. This parameter represents the minimal total sustained force that was exerted to oppose the gravitational force [12].

### Gait analysis

Gait parameters of freely moving mice were measured using the CatWalk gait analysis system (Noldus Information Technology, The Netherlands). The CatWalk instrument consists of a hardware system of a long, enclosed glass walkway plate, illuminated with green light, a high-speed video camera, and a software package for quantitative assessment of animal footprints. A green light emitted by a fluorescent lamp positioned underneath the glass plate is reflected within the glass plate except at points where the mouse paws made contact with the glass plate. It scatters and illuminates the contact area. The intensity of the area of illumination, which is proportional to the exerted pressure, is digitally captured by the video camera connected to a computer that runs the CatWalk software 7.1.

The recordings were carried out when the room was completely dark, except for computer screen. Each mouse was placed individually in the CatWalk walkway and allowed to walk freely, in an unforced manner and traverse from side to side the walkway glass plate. Mouse tracks that were straight without any interruption or hesitation were treated as successful runs. Runs with any wall climbing, grooming, and staying on the walkway were not analyzed. An average number of 3 replicate crossings made by each mouse were recorded. The CatWalk software was used to analyze crossings that had at least five cycles of complete steps. The software automatically labeled all areas containing pixels above the set threshold. These areas were identified and assigned to the respective paws. Analysis of the recording generated a wide range of parameters from which the following gait and coordination parameters were analyzed: Stride length (distance between two consecutive paw placements of the same paw in pixel), swing speed (distance between two consecutive paw placements of the same paw per second), body speed (distance that the animal walks per second) and body speed variation (regularity of body speed, in %) [2, 54, 89].

### Electromyography

Electromyography was performed as previously described [21, 22]. Mice at 10 and 22 month of age were anesthetized with a solution of ketamine/xylazine (100 mg/kg; 5 mg/kg) and electrical activity was recorded using a monopolar needle electrode (diameter 0.3 mm; 9013R0312; Medtronic, Minneapolis, MN, USA) inserted into the tail of the mouse (grounding electrode). Recordings were made with a concentric needle electrode (diameter 0.3 mm; 9013S0011; Medtronic). Electrical activity was monitored in both GA and TA on both legs for at least 2 min. Spontaneous activity was differentiated from voluntary activity by visual inspection. Results were scored as described previously [21, 22].

Compound muscle action potentials (CMAP) were recorded in gastrocnemius muscle as described previously [61]. Briefly, CMAPs were elicited by supramaximal square pulses, of 0.2 ms duration, delivered with a monopolar needle electrode to the sciatic nerve at the sciatic notch level. CMAPs were measured by a monopolar needle electrode inserted in the gastrocnemius, and the system was grounded by subcutaneously inserted monopolar needle electrodes in the back and the tail of the animal. Amplitudes (mV) from the left and right muscle-evoked responses were measured and averaged, resulting in one average CMAP amplitude per animal, which was used for statistical analysis. The latency was measured as the time from the given electrical stimulus to the appearance of a muscle response—the initial CMAP deflection from the baseline.

### Spinal cord motor neurons quantifications

To quantify lower motor neurons, spinal cord cryostat sections of 16 µm were stained with 0.1% Cresyl violet acetate (Certistain^®^, MERK#5235) and anti-ChAT (Millipore, AB144-P; diluted 1:50) followed by biotinylated donkey anti-goat IgG (Jackson, 705-066-147; 1:250) as secondary antibody. The staining was revealed using the ABC kit (Vektor, PK7200; 1:4000), by the avidin–biotin complex immunoperoxidase technique.

Counting of motor neurons was performed in L1–L3 ventral horn in every tenth section for ten sections in total per animal. Total number of motor neurons was counted using ImageJ freeware (http://rsbweb.nih.gov/ij/) after image acquisition at ×20 magnification under the same exposition parameters with a digital camera (Nikon Digital Sight DS-U3). The observer was blinded to the genotype of studied mice.

### RNAseq

Total RNA from spinal cords (including dorsal and ventral roots) of *Fus* ^*ΔNLS/*+^ (*n* = 4) and their control littermates (*n* = 4) were extracted with TRIzol (Invitrogen). RNA quality was measured using the Agilent Bioanalyzer system or RNA screen Tape (Agilent technologies) according to the manufacturer’s recommendations, and processed using the Illumina TruSeq Stranded mRNA Sample Preparation Kit according to the manufacturer’s protocol. Generated cDNA libraries were sequenced using an Illumina HiSeq 2000 sequencer with 4 biological replicates sequenced per condition using single read, 50 cycle runs. Quality of sequencing reads was assessed using FastQC (Babraham Bioinformatics) and then aligned to a mouse reference genome (mm9, UCSC Genome Browser) using TopHat (version v2.0.10). Sequencing yielded, on average, 39 million non-redundant reads per sample with a 55–65% mapping rate. Cufflinks (version v2.1.1) was used to generate transcript abundance for each annotated protein-coding gene as Fragments Per Kilobase of transcript per Million mapped reads (FPKM), and statistical analysis and comparison of FPKM values was calculated using Cuffdiff (version v2.1.1).

### RT-PCR analysis

Spinal cord were harvested, rapidly frozen in liquid nitrogen and stored at −80 °C until analysis. For RT-qPCR, frozen tissues were placed into tubes containing a 5-mm stainless steel bead (Qiagen, Courtaboeuf, France) and 1 ml of Trizol reagent (Invitrogen, Paisley, UK) and homogenized using a Tissue Lyser (Qiagen). RNA was prepared from tissue homogenates following the Trizol manufacturer’s instructions. RNA reverse transcription and SYBR Green real-time PCR assays were performed using the Bio-Rad (Biorad, Marnes la Coquette, France) iCycler kits and protocols. PCR conditions were 3 min at 94 °C, followed by 40 cycles of 45 s at 94 °C and 10 s at 60 °C. Three standard genes: 18S (18S Ribosomal RNA), Pol2 (Polr2 polymerase RNA 2 DNA directed polypeptide A) and Tbp (TATA-box binding protein) were used to compute a normalization factor using Genorm software v3.5 [85]. Primer sequences are provided in Supplementary Table 1.

### Toluidine blue staining

The L4 ventral root sections were stained with toluidine blue to investigate the axon diameter, the degree of demyelination and the myelin pathology among the different groups of animals. Five animals per genotype were analyzed. The ventral roots at the level of L4 were removed, treated with 1% osmium tetroxide, and embedded in Araldite Epon mixture. Semi-thin sections (1.5 µm) were cut, placed on the slides, and oven dried. The slides were stained with 1% toluidine blue solution for 1 min, rinsed with water, dehydrated and mounted. Internal diameter of myelinated axons was measured, and divided by the external diameter to calculate g-ratios. The following abnormalities in myelin were quantified: onion bulbs (as a sign of demyelination and remyelination), demyelinated axons, and abnormal myelin structures including complex or abnormal myelin outfoldings [1, 32]. These quantifications were performed by an observer blinded to the genotype.

### Statistical analysis

For the animal experiments with two groups, the values from each animal were averaged for each genotype group and analyzed by unpaired Student’s *t* test, two-tailed. Comparison of three or four groups was performed using one-way ANOVA and Tukey post hoc test. Data were analyzed by using the Graphics Prism Program (Graph Pad Software, San Diego, CA) and expressed as mean ± SEM (standard error of the mean) and differences were considered significant when *p* ≤ 0.05.

## Results

### Partial cytoplasmic mislocalization of FUS in *Fus*^*ΔNLS/*+^ mice

**Fig. 1:**
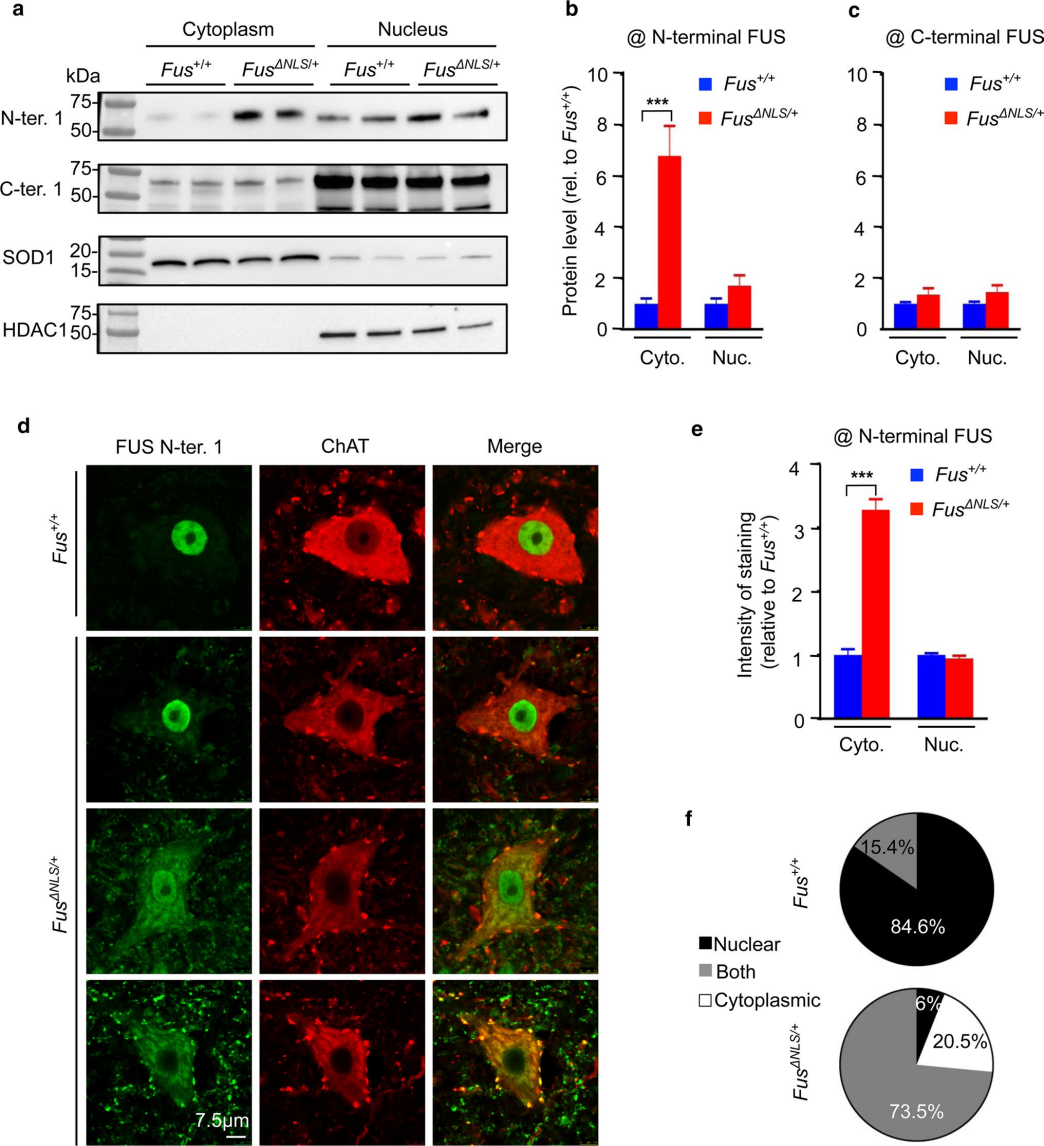
FUS localization in *Fus* ^*ΔNLS*/+^ mice. **a** Immunoblot analysis of FUS protein subcellular localization in spinal cord of 2 *Fus* ^+/+^ and 2 *Fus* ^*ΔNLS*/+^ 4-month-old mice using two different antibodies targeting either the N-terminal part (N-ter. 1) of FUS or the C-terminal (C-ter. 1) NLS. Molecular weight markers are shown on the left. SOD1 and HDAC1 are used as loading controls for cytoplasmic and nuclear protein extracts fractions, respectively. **b**,** c** Quantification of FUS protein levels in cytoplasmic and nuclear fractions from immunoblots for *Fus* ^+/+^ (*blue bars*) and *Fus* ^*ΔNLS*/+^ (*red bars*). *N* = 6. *p < 0.05, ***p < 0.01 by Student’s unpaired *t* test. **d** Double immunostaining for the motoneuronal marker ChAT and FUS (N-terminal part) in the spinal cord ventral horn at 22 months of age. Note the cytoplasmic redistribution of truncated FUS in *Fus* ^*ΔNLS*/+^ mice. *Scale bar* 7.5 µm. **e** Quantification of FUS (N-terminal part) staining intensity in different cellular compartments of motor neuron. *N* = 70 *Fus* ^+/+^, *N* = 68 *Fus* ^*ΔNLS*/+^. ***p < 0.01 by Student’s unpaired *t* test. **f** Distribution of FUS cytoplasmic/nuclear localization in motor neurons. *N* = 70 *Fus* ^+/+^, *N* = 68 *Fus* ^*ΔNLS*/+^

*Fus* ^*ΔNLS/*+^ mice represent the first animal model with a heterozygous mutation in the endogenous *Fus* gene, a similar genetic situation as in ALS-*FUS* patients. *Fus* mRNA levels were modestly increased in spinal cord of *Fus* ^*ΔNLS/*+^ mice suggesting disruption of the normal autoregulatory loop controlling FUS levels (Supplementary Fig. 1a, b), while expression of *Taf15* and *Ewsr1,* the two other FET family members, as well as that of *Tardbp* (encoding TDP-43) was not significantly changed in *Fus* ^*ΔNLS/*+^ mice **(**Supplementary Fig. 1b). Subcellular fractionation of protein extracts from spinal cord followed by western blotting with an antibody recognizing both wild type and ∆NLS FUS protein (N-ter 1) yielded a robust FUS signal in cytoplasmic fractions from *Fus* ^*ΔNLS/*+^ spinal cord, but not from wild-type littermate spinal cord (Fig. 1a, b). Contrastingly, western blotting using an antibody specific for the C-terminal NLS of FUS, and thus unable to recognize the mutant FUS protein, did not show increased cytoplasmic levels (Fig. 1c), demonstrating that FUS protein produced from the wild-type allele remains mostly nuclear. Nuclear FUS levels were not altered on immunoblots using either of the two antibodies (Fig. 1a–c). Consistently, increased cytoplasmic FUS staining was observed in *Fus* ^*ΔNLS/*+^ motor neurons using double immunofluorescence with FUS and choline acetyltransferase (ChAT) antibodies (Fig. 1d). Quantification of fluorescence signals in individual motor neurons showed that the cytoplasmic staining of FUS was substantially elevated in *Fus* ^*ΔNLS/*+^ motor neurons (Fig. 1e). The large majority (85%) of *Fus* ^+*/*+^ motor neurons demonstrated an exclusive localization of FUS in the nucleus, while most (74%) *Fus* ^*ΔNLS/*+^ motor neurons showed mixed cytoplasmic and nuclear localization. Notably, 21% of motor neurons showed exclusively cytoplasmic FUS accumulation accompanied by complete nuclear clearance of FUS protein (Fig. 1f). We did not observe large cytoplasmic FUS aggregates associated with FUS nuclear clearance.

### *Fus*^*ΔNLS/*+^ mice recapitulate pathological hallmarks of ALS-*FUS*

**Fig. 2:**
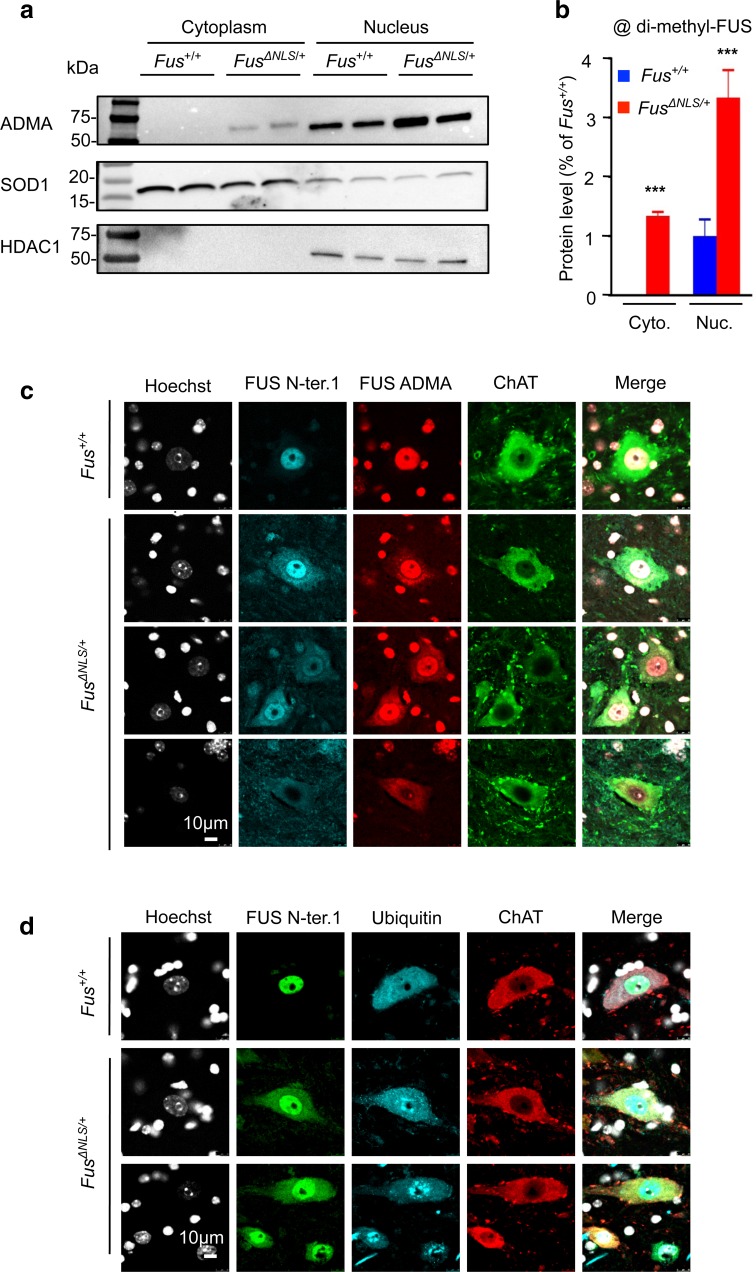
Subcellular redistribution of asymmetrically arginine dimethylated (ADMA) FUS. **a** Representative immunoblots on cytoplasmic and nuclear fractions of protein extracts from spinal cord of *Fus* ^+/+^ and *Fus* ^*ΔNLS*/+^ mice, using an antibody recognizing asymmetrically arginine dimethylated FUS (ADMA-FUS). HDAC1 is used as a loading control for nuclear fractions and SOD1 for cytoplasmic fractions. Molecular weight markers are shown on the* left*. **b** Quantification of ADMA-FUS protein levels in cytoplasmic and nuclear fractions from immunoblots for *Fus* ^+/+^ (*blue bars*) and *Fus* ^*ΔNLS*/+^ (*red bars*). *N* = 6. *p < 0.05, ***p < 0.01 by Student’s unpaired *t* test. **c** Triple immunostaining for the motoneuronal marker ChAT (*green*), for FUS (N-terminal part) (*cyan*) and for ADMA-FUS (*red*) in the spinal cord ventral horn. *Scale bar* 10 µm. **d** Triple immunostaining with antibodies to the N terminus of FUS (*green*), ChAT (*red*) and pan-Ubiquitin (*cyan*), showing diffuse cytoplasmic and nuclear punctate aggregates within motor neurons with relocated FUS. *Scale bar* 10 μm

In human ALS-*FUS* patients, FUS is asymmetrically dimethylated at arginine residues (ADMA), and this modified form of FUS is found in FUS-positive inclusions [19, 75, 81]. In contrast, unmethylated FUS and monomethylated FUS, but not ADMA-FUS, accumulates in cytoplasmic inclusions of FTLD-FUS patients [19, 75]. ADMA-FUS can be readily identified using an antibody specific to the ADMA RGG3 domain of FUS [19]. Interestingly, ADMA-FUS was strongly increased in both nuclear and cytoplasmic fractions of *Fus* ^*ΔNLS/*+^ spinal cord (Fig. 2a, b). Furthermore, triple immunolabeling using antibodies against FUS, ADMA-FUS and ChAT revealed a pattern of ADMA-FUS subcellular distribution similar to truncated FUS (Fig. 2c). ALS-*FUS* patients also develop ubiquitin and p62 pathology [43]. While we did not observe p62 inclusions (Supplementary Fig. 3), we observed cytoplasmic and nuclear ubiquitin pathology in motor neurons of *Fus* ^*ΔNLS/*+^ mice (Fig. 2d). Cytoplasmic ubiquitin pathology was also observed using a K63 linkage-specific ubiquitin antibody (Supplementary Fig. 2). Ubiquitin inclusions were occasionally present in motor neurons with mixed nuclear/cytoplasmic FUS localization and systematically present in motor neurons showing complete nuclear FUS clearance (Fig. 2d). Importantly, FUS and ubiquitin stainings did not systematically overlap, suggesting that *Fus* ^*ΔNLS/*+^ motor neurons do not develop ubiquitin-positive FUS inclusions. Thus, *Fus* ^*ΔNLS/*+^ mice and ALS-*FUS* patients develop partially similar pathology, with cytoplasmic accumulation of methylated FUS and ubiquitin pathology but no large FUS aggregates.

### Cytoplasmic mislocalization of FUS leads to a mild motor deficit in *Fus*^*ΔNLS/*+^ mice

We next investigated whether cytoplasmic accumulation of mutant FUS triggers ALS-like motor symptoms during the lifespan of heterozygous knock-in animals. To this aim, we longitudinally followed *Fus* ^*ΔNLS/*+^ male mice and their *Fus* ^+*/*+^ wild-type littermates until 2 years of age. Animals were weekly monitored for general health, neurological symptoms, body weight, grip strength and accelerating rotarod performance. Until 22 months of age, when mice were killed, expression of mutant FUS was neither associated with important weight loss nor with development of paralysis (Supplementary Fig. 4a–c). Although grip test and rotarod are among the most commonly used tests to assess motor function in mice [70], they often lack the sensitivity needed to detect subtle alterations in the motor system and should be complemented by additional tests to evaluate motor function in *Fus* ^*ΔNLS/*+^mice.

**Fig. 3:**
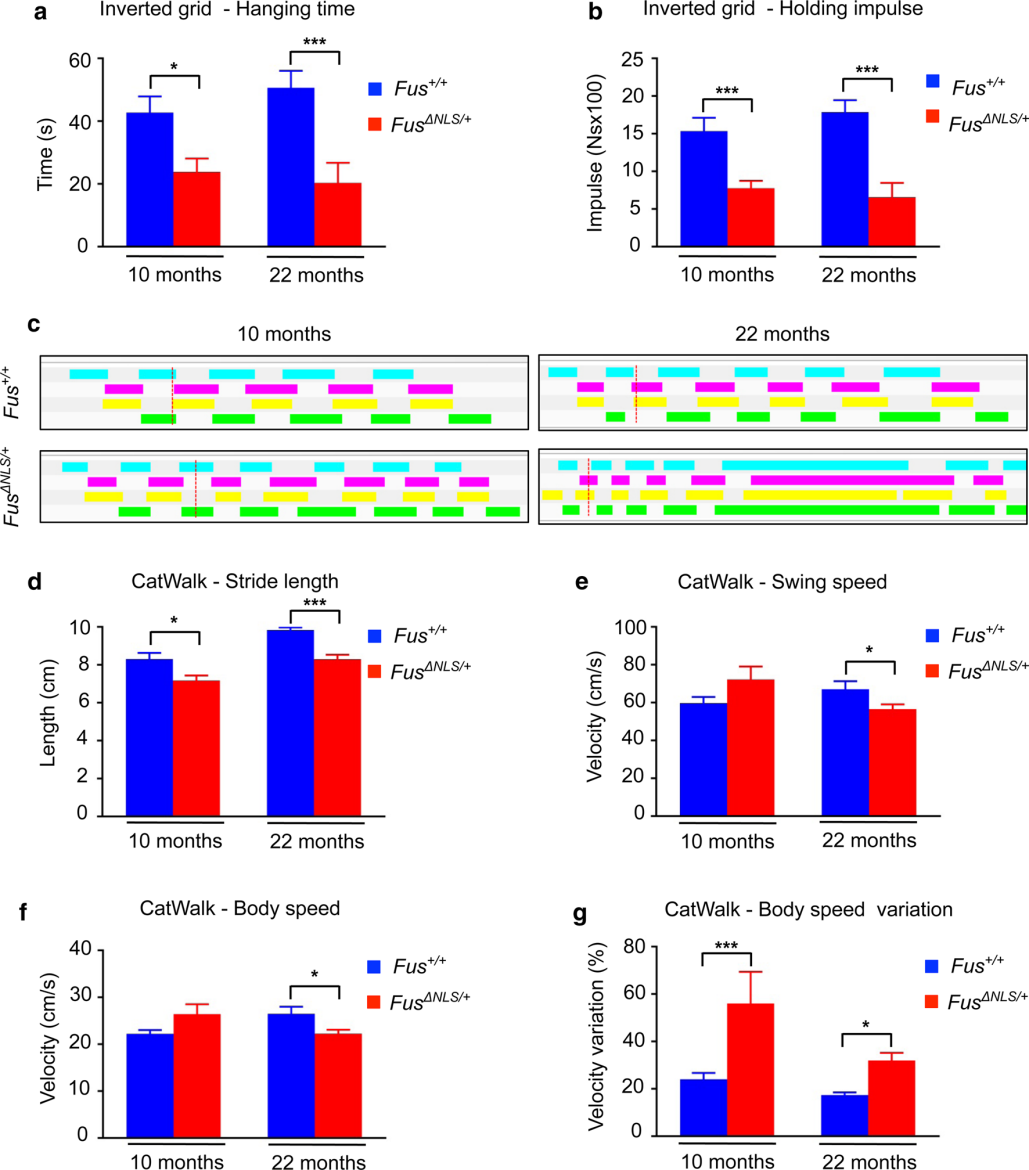
*Fus* ^*ΔNLS*/+^ mice display a mild motor deficit. Age-dependent changes in the mean hanging time (s) (**a**) and holding impulse (N s) (**b**) in the four-limb wire inverted grid test in *Fus* ^+/+^ and *Fus* ^*ΔNLS*/+^ mice. *N* = 7 for 10 months; *N* = 5 for 22 months. *p < 0.05, ***p < 0.01 as compared to *Fus* ^+/+^ by Student’s unpaired t test. **c** Representative gait patterns of *Fus* ^+/+^and *Fus* ^*ΔNLS*/+^ mice at 10 months (*left panels*) and 22 months (*right panels*) of age. The panels show the digitized prints with* colorful* phase lags representing the stance phase duration of each individual paw in a single-step cycle. **d**–**g** Gait changes and variability in *Fus* ^*ΔNLS*/+^mice. Stride length (**d**, distance between successive placements of the same paw in cm); swing speed (**e**, distance traveled by one paw per second), body speed (**f**, distance traveled by the animal per second, in cm/s), and body speed variation (**g**, regularity of body speed in %), are shown. *N* = 3 for 10 months; *N* = 5 for 22 months. All graphs show the overall sample means and standard errors at various ages (10 months; 22 months) for *Fus* ^+/+^(*blue bars*) and *Fus* ^*ΔNLS*/+^ (*red bars*) mice. *p < 0.05, ***p < 0.01 as compared to Fus^+/+^ by Student’s unpaired *t* test

Indeed, despite normal performance on rotarod and grip strength, *Fus* ^*ΔNLS/*+^ mice displayed a significantly shorter hanging time in an inverted grid test (Fig. 3a), both at 10 and 22 months of age. Holding impulse, which represents the total sustained force exerted by the mouse to oppose the gravitational force [12], was also significantly decreased in *Fus* ^*ΔNLS/*+^ mice (Fig. 3b). The evaluation of gait performance by CatWalk analysis further confirmed this motor defect. *Fus* ^*ΔNLS/*+^ mice demonstrated an irregular walking pattern characterized by phases of fast walking interrupted with stance phases (Fig. 3c). We also observed a reduction of hind limb stride length (Fig. 3d) associated with an increase in body speed variation (i.e., the variation in the speed of the walking mouse) for both ages compared to the control mice (Fig. 3g). In addition, 22-month-old *Fus* ^*ΔNLS/*+^ mice showed impaired swing and body speed (Fig. 3e–f). Thus, expression of mutant FUS at a physiological level is associated with partial cytoplasmic mislocalization of the protein and a mild motor deficit in mice.

### Cytoplasmic mislocalization of FUS drives age-dependent, progressive motor neuron degeneration

**Fig. 4:**
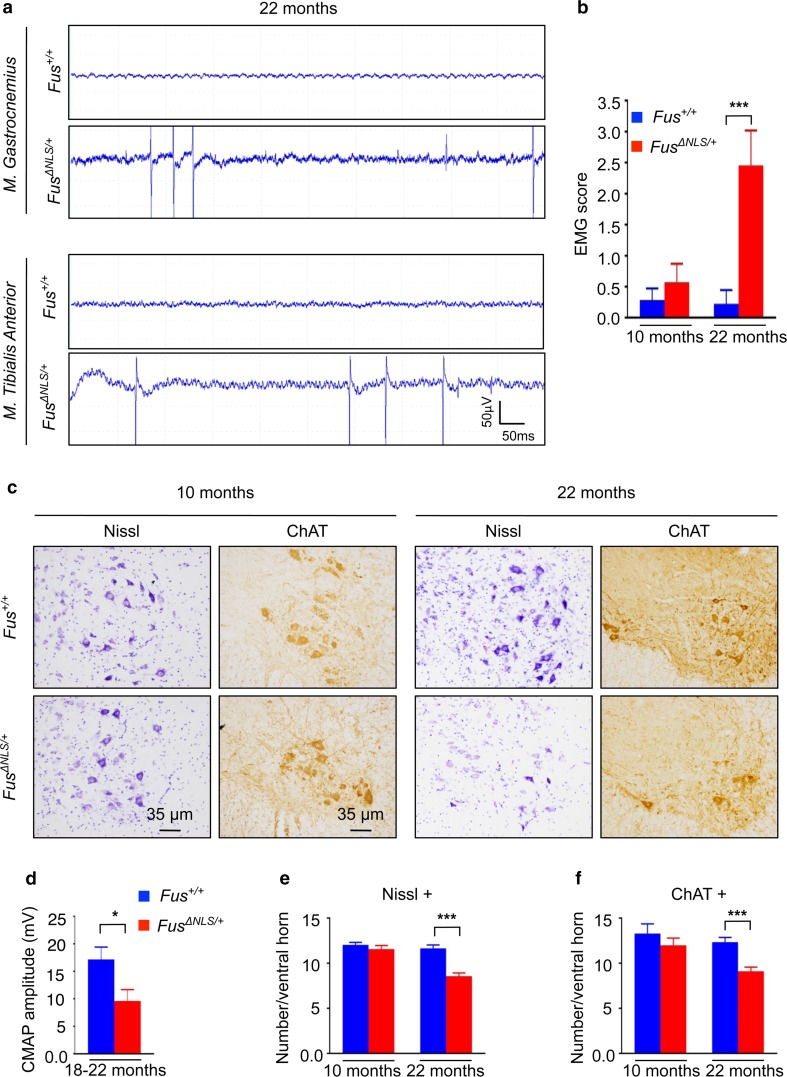
Muscle denervation and progressive degeneration of spinal motor neurons in *Fus* ^*ΔNLS*/+^ mice. **a** Representative electromyograms of *Fus* ^*ΔNLS*/+^ mice in *gastrocnemius* and *tibialis anterior* muscles. Note the presence of typical spontaneous denervation activities (fibrillation potentials) in *Fus* ^*ΔNLS*/+^ mice. *Scale bars* 50 ms and 50 µV. **b** Graph showing EMG scores for *Fus* ^+/+^ (*blue bars*) and *Fus* ^*ΔNLS*/+^ (*red bars*) mice. Note that a significant difference was only detected for 22-month-old animals. ***p < 0.01 as compared to *Fus* ^+/+^; *N* = 7 for 10 months; *N* = 9 for 22 months; Student’s unpaired *t* test. **c** Representative images of Nissl and ChAT staining of spinal cord ventral horn of 10-month-old (*left panels*) and 22-month-old (*right panels*) *Fus* ^+/+^ and *Fus* ^*ΔNLS*/+^ animals. In the 22-month-old *Fus* ^*ΔNLS*/+^ mice degenerative changes (shrinking, chromatolysis) and loss of motor neurons occur. *Scale bars* 35 μm. **d** Compound muscle action potential (CMAP) amplitude. *p < 0.05 as compared to *Fus* ^+/+^; *N* = 10 *Fus* ^+/+^, *N* = 9 *Fus* ^*ΔNLS*/+^. Student’s unpaired *t* test. Bar graphs showing means and standard errors of Nissl+ **(e)** and ChAT+ **(f)** motor neuron number in the ventral horn of the spinal cord at 10 months and 22 months for *Fus* ^+/+^ (*blue bars*) and *Fus* ^*ΔNLS*/+^ (*red bars*) mice. ***p < 0.01 as compared to *Fus* ^+/+^; *N* = 3 for 10 months; *N* = 6 for 22 months; Student’s unpaired *t* test

To determine whether this mild motor phenotype could be due to an underlying motor neuron disease, we performed electromyography analysis (EMG) on *Fus* ^+*/*+^ and *Fus* ^*ΔNLS/*+^ mice at 10 and 22 months of age. We did not observe stereotypical denervation-related electrical activities in gastrocnemius (GA) or tibialis anterior (TA) muscle of 10-month-old *Fus* ^+*/*+^ and *Fus* ^*ΔNLS/*+^ mice. However, 22-month-old *Fus* ^*ΔNLS/*+^ mice showed typical fibrillation and fasciculation potentials in both muscles (Fig. 4a). Consistent with qualitative observations, a quantitative analysis of the EMG recordings demonstrated a significantly increased frequency of abnormal potentials in 22-month-old *Fus* ^*ΔNLS/*+^ mice, but not at 10 months of age (Fig. 4b) and the amplitude of compound muscle action potentials (CMAP) was decreased in 18- to 22-month-old *Fus* ^∆NLS/+^ mice (Fig. 4d).

Importantly, the abnormal electrical activity in *Fus* ^*ΔNLS/*+^ mice was accompanied by degeneration of motor neurons in the lumbar spinal cord of 22-month-old *Fus* ^*ΔNLS/*+^ mice, as evaluated using either Nissl staining or immunostaining for ChAT (Fig. 4c). Quantitative analysis revealed that the number of motor neurons was reduced by ~30% in 22-month-old *Fus* ^*ΔNLS/*+^ mice as compared to *Fus* ^+*/*+^ mice (Fig. 4e–f). Importantly, the number of lumbar spinal cord motor neurons was not altered at 10 months of age, indicating that the pathological process is progressive. Thus, partial cytoplasmic mislocalization of FUS triggers late-onset progressive motor neuron loss associated with a mild motor deficit.

### Reduced levels of FUS do not lead to motor neuron degeneration

**Fig. 5:**
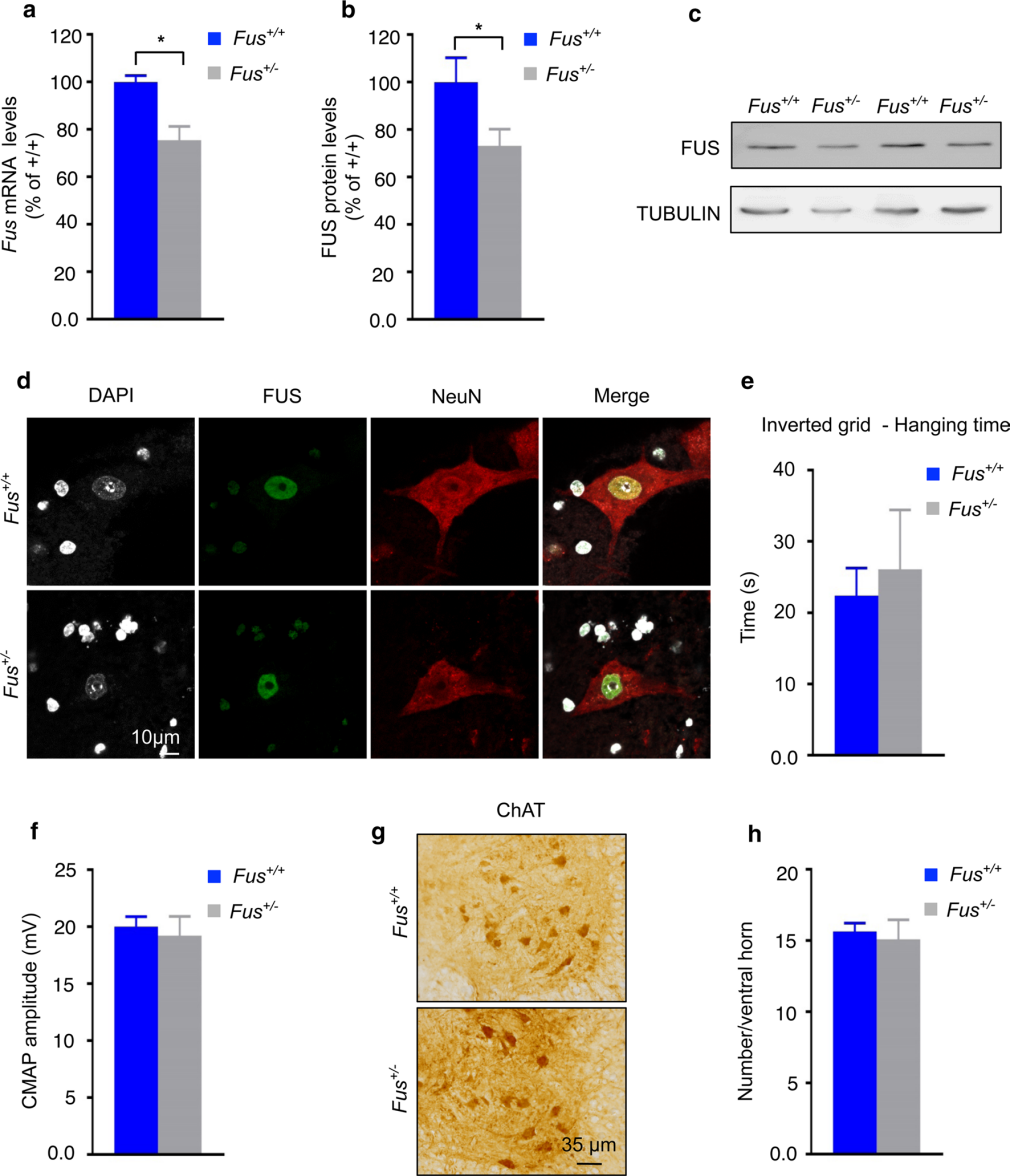
Lack of motor neuron disease in *Fus* ^+*/*−^ mice. **a** Expression levels of *Fus* mRNA in spinal cord. *Fus* mRNA levels were significantly reduced in *Fus* ^+*/*−^ mice as revealed by quantitative real-time PCR analysis. *N* = 7 *Fus* ^+/+^, *N* = 9 *Fus* ^+*/*−^. *p < 0.05 by Student’s unpaired *t* test. **b** Quantification of FUS protein levels from immunoblots showed a lower amount of FUS in spinal cord of *Fus* ^+*/*−^ mice. *N* = 7 *Fus* ^+/+^, *N* = 8 *Fus* ^+*/*−^. *p < 0.05 by Student’s unpaired *t* test. **c** Representative immunoblot for FUS on protein extracts from spinal cord of 100-week-old mice. TUBULIN was used as loading control. **d** Immunostaining for the neuronal marker NeuN and FUS on the spinal cord ventral horn of 100-week-old *Fus* ^+*/*+^ and *Fus* ^+*/*−^ mice. Note preserved nuclear localization of FUS in *Fus* ^+*/*−^ mice. **e** Mean hanging time in the four-limb wire inverted grid test of *Fus* ^+/+^ and *Fus* ^+*/*−^ mice. *N* = 4 *Fus* ^+/+^, *N* = 3 *Fus* ^+*/*−^. *p* = not significant (NS) by Student’s unpaired *t* test. **f** *Bar graphs* showing means and standard errors for compound muscle action potential (CMAP) amplitude. No difference was observed between groups. *N* = 6 *Fus* ^+/+^, *N* = 5 *Fus* ^+*/*−^. *p* = NS by Student’s unpaired *t* test. **g** Representative images of ChAT immunostaining on spinal cord ventral horn. *Scale bar* 35 μm. **h** Quantification of the number of motor neurons per spinal cord ventral horn in *Fus* ^+/+^ and *Fus* ^+*/*−^ mice. The number of ChAT+ motor neurons was not altered in *Fus* ^+*/*−^ mice. *N* = 6 *Fus* ^+/+^, *N* = 5 *Fus* ^+*/*−^. *p* = NS by Student’s unpaired *t* test

We next investigated whether partial loss of nuclear FUS function might contribute to the observed phenotypes of *Fus* ^*ΔNLS/*+^ mice. To determine whether a mild loss of FUS function could be sufficient to trigger motor neuron degeneration, we longitudinally followed *Fus* ^+/−^ mice [68]. At the age of 23 months, *Fus* transcript levels were reduced by 25% in spinal cord of *Fus* ^+/−^ mice (Fig. 5a). Consistently, western blot analysis revealed reduced FUS protein levels in the spinal cord of these mice (Fig. 5b, c). These reduced FUS expression levels did not alter the subcellular localization of FUS in the spinal cord (Fig. 5d). Up to the age of 23 months, the performance of *Fus* ^+/−^ mice in the inverted grid test (Fig. 5e) was not different from *Fus* ^+/+^ control mice, as well as their grip strength and body weight (data not shown). Consistently, *Fus* ^+/−^ mice did neither show denervation potentials in EMG nor decreased CMAP (Fig. 5f). Finally, normal numbers of spinal motor neurons were found at 23 months of age (Fig. 5g, h). Thus, *Fus* reduction is not sufficient to trigger the motor neuron defects observed in *Fus* ^*ΔNLS/*+^ mice.

### Cre-dependent reversal of the *Fus* mutation in motor neurons largely prevents FUS mislocalization

We then sought to determine whether motor neuron degeneration in *Fus* ^*ΔNLS/*+^ mice was dependent on the mislocalization of FUS in motor neurons themselves. To this aim, we took advantage of the presence of loxP sites on the ∆NLS allele, allowing us to restore a normal protein in specific cell types upon CRE recombination [68]. *Fus* ^*ΔNLS/*+^ mice were bred with mice expressing the CRE recombinase from the endogenous *Chat* locus, which leads to CRE recombinase activity in virtually all cholinergic neurons [65, 67]. In double transgenic *Fus* ^*ΔNLS/*+^/*Chat*-CRE mice, the cytoplasmic accumulation of FUS was rescued, although not completely (Supplementary Fig. 5a), and the proportion of motor neurons with complete nuclear FUS clearance was reduced to similar levels as in *Fus* ^+*/*+^ mice (Supplementary Fig. 5b–d). Consistently, immunoreactivity for ADMA-FUS was barely detectable in the cytoplasm of motor neurons of *Fus* ^*ΔNLS/*+^/*Chat*-CRE mice (Supplementary Fig. 6). Thus, motor neuron-selective reversal of the *Fus* mutation rescues FUS mislocalization in motor neurons of *Fus* ^*ΔNLS/*+^ mice.

### Reversal of the *Fus* mutation in motor neurons prevents motor neuron degeneration and delays motor deficits

**Fig. 6:**
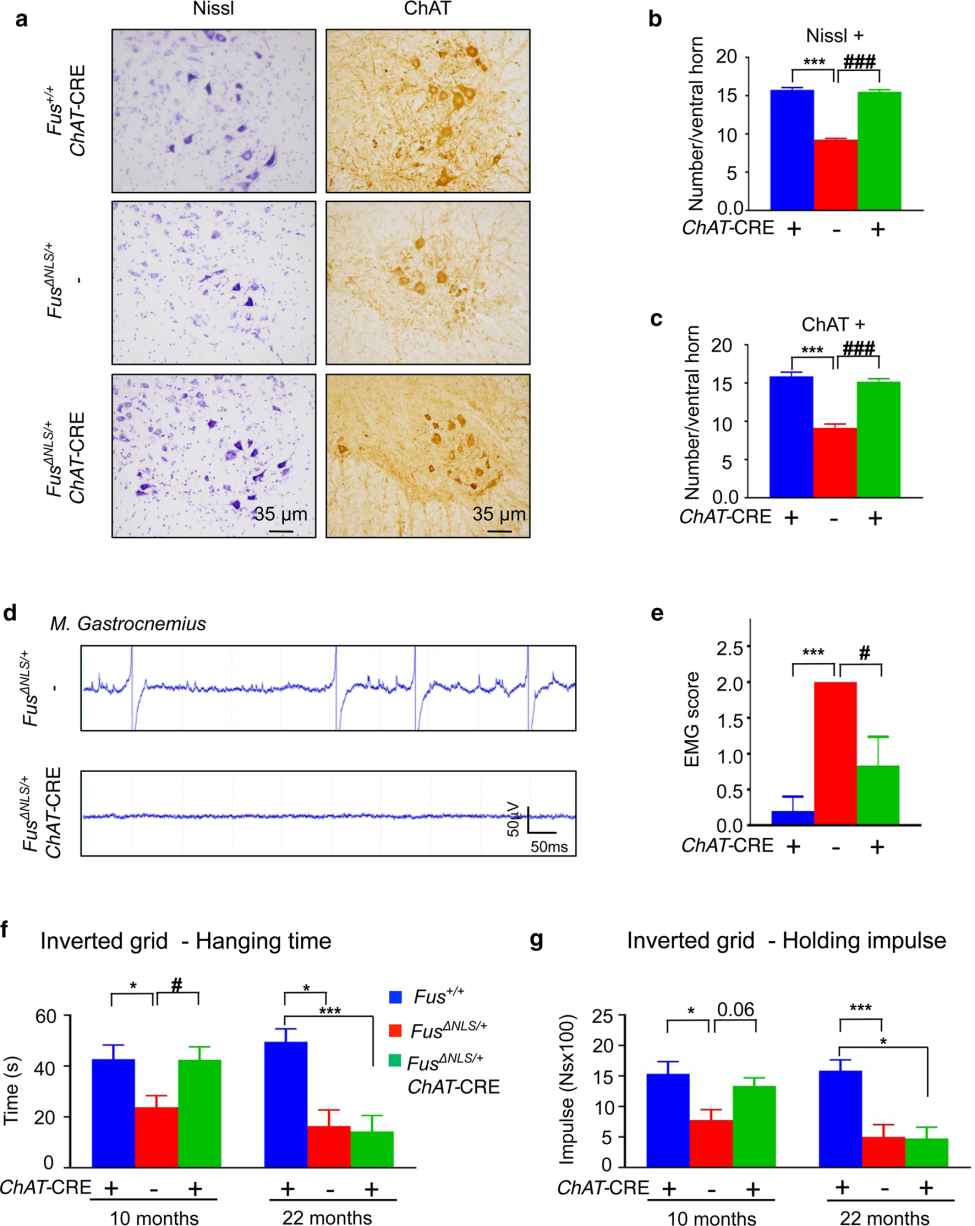
Motor neuron-selective reversal of the *Fus* ^*ΔNLS*^ allele to wild type delays but does not prevent *Fus* ^*ΔNLS/*+^ motor phenotypes. **a** Representative images of spinal cord ventral horn of *Fus* ^+/+^/*ChAT*-CRE, *Fus* ^*ΔNLS/*+/−^ and *Fus* ^*ΔNLS/*+^/*ChAT*-CRE mice at 22 months stained with Nissl (*left panels*) or anti-choline acetyl transferase (ChAT, *right panels*). **b**,** c** Quantification of motor neurons per spinal cord ventral horn. The number of Nissl+ **(b)** and ChAT+ **(c)** motor neurons is rescued in *Fus* ^*ΔNLS/*+^/*ChAT*-CRE mice while significantly reduced in *Fus* ^*ΔNLS/*+/−^ mice. *N* = 5 per genotype; ***p < 0.01 versus *Fus* ^+/+^/*ChAT*-CRE, ^###^ *p* < 0.01 versus *Fus* ^*ΔNLS/*+^/*ChAT*-CRE; one-way ANOVA followed by Tukey post hoc test. In all graphs genotypes are represented as *Fus* ^+/+^/*ChAT*-CRE (*blue bars*), *Fus* ^*ΔNLS/*+/−^ (*red bars*) and *Fus* ^*ΔNLS/*+^/*ChAT*-CRE (*green bars*). **d** EMG recording traces in gastrocnemius muscle of 22-month-old animals. Note the absence of typical spontaneous denervation activities in *Fus* ^*ΔNLS/*+^/*ChAT*-CRE versus *Fus* ^*ΔNLS/*+/−^ mice. *Scale bars* 50 ms and 50 µV. **e** EMG score showing significantly decreased spontaneous activity in *Fus* ^*ΔNLS/*+^/*ChAT*-CRE as compared to *Fus* ^*ΔNLS/*+/−^ in 22-month-old animals. *N* = 5 *Fus* ^+/+^/*ChAT*-CRE, *N* = 7 *Fus* ^*ΔNLS/*+/−^ and *N* = 6 *Fus* ^*ΔNLS/*+^/*ChAT*-CRE. ***p < 0.01 versus *Fus* ^+/+^/*ChAT*-CRE; ^#^ *p* < 0.05 versus *Fus* ^*ΔNLS/*+^/*ChAT*-CRE; one-way ANOVA followed by Tukey post hoc test. Inverted grid test mean hanging time **(f)** and holding impulse **(g)**. *N* = 7–8 for 10 months; *N* = 5–7 for 22 months. *p < 0.05, ***p < 0.01 versus *Fus* ^+/+^/*ChAT*-CRE; ^#^ *p* < 0.05 versus *Fus* ^*ΔNLS/*+^/*ChAT*-CRE; one-way ANOVA followed by Tukey post hoc test

The presence of a *Chat*-CRE allele in *Fus* ^*ΔNLS/*+^ mice was sufficient to fully restore motor neuron counts to similar numbers as in *Fus* ^+*/*+^ mice at 22 months of age (Fig. 6a–c). Consistently, *Fus* ^*ΔNLS/*+^/*Chat*-CRE mice did not develop the EMG abnormalities found in their *Fus* ^*ΔNLS/*+^ littermates and their EMG score did not significantly differ from *Fus* ^+*/*+^ mice (Fig. 6d–e). While littermate *Fus* ^*ΔNLS/*+^ mice displayed a significantly shorter hanging time and shorter holding impulse than *Fus* ^+*/*+^ mice in the inverted grid test at both 10 and 22 months of age (Figs. 3a, b, 6f, g), *Fus* ^*ΔNLS/*+^/*Chat*-CRE mice displayed motor performance similar to *Fus* ^+*/*+^ mice at 10 months of age (Fig. 6f, g). At 22 months of age, however, *Fus* ^*ΔNLS/*+^/*Chat*-CRE mice displayed significantly impaired motor performance as compared to *Fus* ^+*/*+^ mice, leading to a similar motor deficit as *Fus* ^*ΔNLS/*+^ mice. Thus, the selective reversal of the *Fus* mutation in motor neurons is sufficient to fully rescue motor neuron degeneration, even at older ages, while the motor deficits are delayed but not prevented.

### Oligodendrocytic alterations in *Fus*^*ΔNLS/*+^ spinal cord

**Fig. 7:**
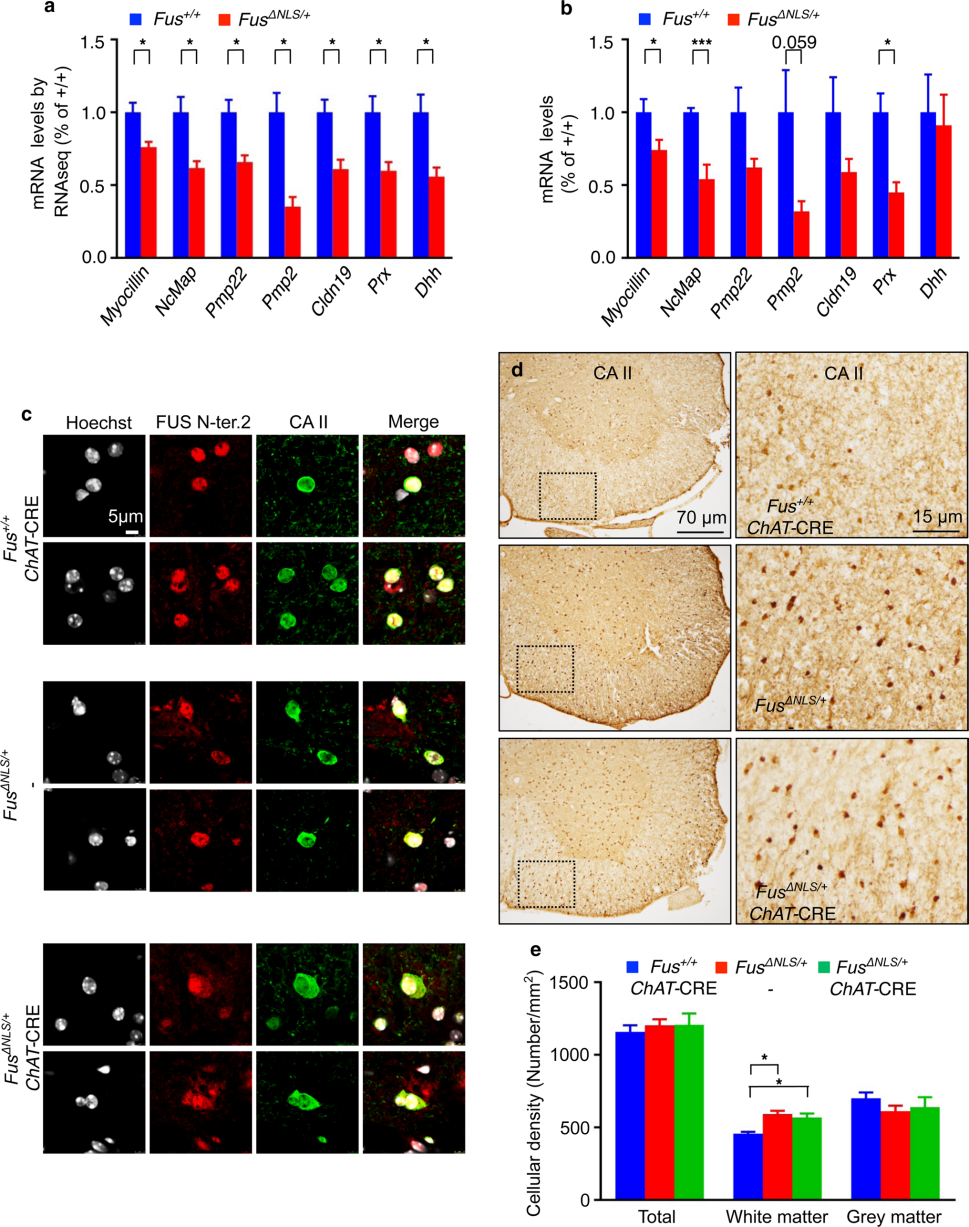
Axonal and myelin abnormalities in *Fus* ^*ΔNLS/*+^ mice. **a**, **b** Normalized expression of myelin-related genes (*Myocilin, NcMap, Pmp22, Pmp2, Cldn19, Prx, Dhh)* in *Fus* ^*ΔNLS/*+^ compared to their control littermates based on FPKM from RNAseq (*a*, *n* = 4) or RT quantitative PCR (*b*, *n* = 6–7). *Error bars* represent SEM. *p < 0.05, ***p < 0.01; Student’s *t* test. **c** Double immunolabeling for the oligodendrocyte marker carbonic anhydrase II (CAII) (*green*) and FUS (N-terminal part) (*red*) in the spinal cord. *Scale bar* 5 µm. **d** Representative images of spinal cord ventral horn of *Fus* ^+/+^/*ChAT*-CRE, *Fus* ^*ΔNLS/*+^ and *Fus* ^*ΔNLS/*+^/*ChAT*-CRE mice at 22 months stained with anti-carbonic anhydrase II showing increased numbers of oligodendrocytes. *Scale bars*: *left column* 70 µm; *right column* 15 µm. **e** Oligodendrocyte density in whole spinal cord ventral quadrant, and separately in white and grey matter. Note increased numbers in white matter in 22-month-old *Fus* ^*ΔNLS/*+/−^, not rescued in *Fus* ^*ΔNLS/*+^/*ChAT*-CRE mice. *N* = 4 animals per genotype. ***p < 0.01 versus *Fus* ^+/+^/*ChAT*-CRE; one-way ANOVA followed by Tukey post hoc test

To evaluate the molecular mechanisms underlying the phenotypes in *Fus* ^*ΔNLS/*+^ mice, we performed RNAseq on spinal cord RNA extracts from 22-month-old *Fus* ^*ΔNLS/*+^ and control littermates. Among the genes showing differential expression, several of them encoded for proteins related to myelination (Fig. 7a). Indeed, mRNA levels of *myocilin, Ncmap, Pmp2, Pmp22, Cldn19* and *Prx*, were all downregulated in RNAseq from *Fus* ^*ΔNLS/*+^ mice (Fig. 7a), and this was confirmed using RT-qPCR on samples obtained from an independent cohort of mice (Fig. 7b). For instance, *Myocilin* is required for peripheral myelination [46], as is *Pmp2* [94] and *Prx* [25]. *Pmp22* [33] and *Prx* [15] are involved in the morphology of myelinating Schwann cells. Interestingly, we did not observe altered expression of other major myelin genes such as *Mbp, Plp1* or *Abca1* (Supplementary Fig. 7). Increased cytoplasmic FUS staining was observed in *Fus* ^*ΔNLS/*+^ oligodendrocytes using double immunofluorescence for FUS and oligodendrocyte specific markers CNPase and carbonic anhydrase II (Fig. 7c; supplementary Fig. 8). Oligodendrocytes were more numerous in ventral horn white matter of *Fus* ^*ΔNLS/*+^ mice as compared to *Fus* ^+/+^ mice (Fig. 7d, e), and this was not reverted in *Fus* ^*ΔNLS/*+^/*Chat*-CRE mice. Together with RNAseq results, these data suggest that FUS mislocalization leads to defective oligodendrocyte physiology, independently of motor neuron involvement. To understand whether these defects could translate into abnormal myelination of motor axons, we studied the morphology of the ventral roots that collect all motor axons exiting from the spinal cord at 22 months of age. Consistent with a defect in myelination, g-ratio of ventral root axons were modestly decreased for the smaller axonal calibers (Supplementary Fig. 9a). Furthermore, motor axons of *Fus* ^*ΔNLS/*+^ mice showed a distribution shifted towards smaller caliber (Supplementary Fig. 9b), and this axonal defect was not rescued in *Fus* ^*ΔNLS/*+^/*Chat*-CRE mice (Supplementary Fig. 9b, c). Moreover, there were fewer axons showing normal myelination in ventral roots of both *Fus* ^*ΔNLS/*+^ and *Fus* ^*ΔNLS/*+^/*Chat*-CRE mice as compared to control littermates (Supplementary Fig. 9d), and thus a higher frequency of typical features of myelination defects in both *Fus* ^*ΔNLS/*+^ groups. Last, consistent with a myelin defect, the latency of CMAP was increased in *Fus* ^*ΔNLS/*+^ muscles (Supplementary Fig. 9e). These results indicate that the function of myelinating cells, both in spinal cord and in the periphery, is altered in *Fus* ^*ΔNLS/*+^ mice, what may contribute to the observed motor deficits in aged mice.

## Discussion

In this study we show that a heterozygous mutation in the endogenous murine *Fus* gene, that is similar to the most severe mutations in juvenile ALS, partially recapitulates ALS-*FUS* pathology and triggers mild progressive ALS-like symptoms. We demonstrate that this *Fus* mutation is associated with motor neuron degeneration through FUS mislocalization in motor neurons, while axonal damage and demyelination occur independent of mutant FUS expression in motor neurons.

The generation of faithful animal models of neurodegenerative diseases is a long-standing aim of the scientific community. Recently, heterozygous knock-in mouse models of Alzheimer’s disease were shown to display mild but significant behavioral abnormalities, overcoming most of the artificial phenotypes observed in classical transgenic AD mouse models [66]. Similar artificial phenotypes are also confounding analysis in most currently used transgenic mouse models of ALS [5, 23, 30]. Indeed, the vast majority of described ALS mice expressing either mutant SOD1 [29], mutant TDP-43 [90] or mutant FUS [64, 73], are multi-copy transgenic lines, with poorly documented sites of transgene insertion. Moreover, overexpression of the wild type forms of SOD1 [26], TDP-43 [78, 91, 92] or FUS [55] generally leads to similar, if not exacerbated, symptoms as compared to expression of the ALS-linked mutations, casting doubts on their relevance as faithful animal models of the disease. Recently, Sharma and collaborators generated mice with targeted expression of wild type or mutant FUS from the *Tau* locus [72]. These mice express physiological levels of FUS, yet under the control of the *Tau* promoter which is more active in neurons than in other cell types of the CNS. In *Fus* ^*ΔNLS/*+^ mice, the endogenous murine *Fus* gene carries the mutation on a single allele thus providing the unique opportunity to study the effects of an ALS-like mutation in an authentic genetic context. The genetic construct used here features a poly-adenylation cassette that precludes the inclusion of regulatory elements present in the endogenous 3′UTR of the *Fus* mRNA. Since the 3′UTR of *Fus* mRNA has been found important for FUS autoregulation [17], this could in principle result in altered autoregulation of FUS. However, exon 7 and adjacent introns that are primarily involved in *Fus* autoregulation are conserved in this model [95].

*Fus* ^*ΔNLS/*+^ mice replicated a number of the pathological hallmarks observed in ALS-*FUS* patients. Similar to ALS-*FUS* patients or iPSC-derived cells of these patients, the FUS protein was partially mislocalized to the cytoplasm [36, 38, 45, 48, 51] in a dimethylated form [19, 75]. Consistent with the heterogeneity observed in human cases, *Fus* ^*ΔNLS/*+^ motor neurons displayed various stages of FUS mislocalization [51, 52], and a subset of them showed complete nuclear FUS clearance. Importantly, not all pathological features of ALS-*FUS* were reproduced, suggesting that *Fus* ^*ΔNLS/*+^ mice recapitulate early stages of disease. For instance, we did not observe neuronal or glial large cytoplasmic inclusions of FUS [43, 51], even in cells showing complete nuclear clearance of FUS. This is similar to mice expressing mutant FUS from the TAU locus [72]. Although FUS does not spontaneously form large pathological aggregates, we cannot exclude that the biochemical properties of FUS could be altered in these mice. For instance, a proportion of the FUS protein could become insoluble and/or its repertoire of binding partners could be modified [6, 86]. Further work in *Fus* ^*ΔNLS/*+^ mice should clarify the biochemical consequences of FUS truncation and mislocalization.

There was significant ubiquitin pathology but no p62 pathology. Such an early pathological stage is consistent with the observed slowly progressive motor neuron loss, mild motor symptoms, and a presumably unaltered mouse lifespan at least until 22 months of age. Several factors could explain why *Fus* ^*ΔNLS/*+^ mice did not progress to the full-blown pathological and behavioral features of ALS-*FUS*. First, the genetic background of mice influences ALS-related disease course [31, 53]. Second, it cannot be excluded that additional hits, either environmental or genetic, are necessary for the progression of ALS-*FUS*, as previously postulated [18]. Third, the life span of mice might simply be too short to develop full-blown ALS, as in humans the disease becomes symptomatic after several decades.

Both ALS-*FUS* patients and *Fus* ^*ΔNLS/*+^ mice carry one mutant copy of the FUS gene, and comparing *Fus* ^*ΔNLS/*+^ mice with *Fus* ^+*/*−^ mice allowed us to provide definitive evidence that gain of function is required to cause ALS-*FUS*. Indeed, *Fus* ^+*/*−^ mice did not show motor neuron loss or motor symptoms in contrast to *Fus* ^*ΔNLS/*+^ mice. This is consistent with the absence of motor phenotypes in *Fus* knock-out mice, showing that even the complete absence of the FUS protein is not sufficient to trigger motor neuron degeneration [44, 68, 72]. Our results are consistent with recently published studies documenting that cytoplasmic FUS accumulation is the only necessary toxic event to trigger motor neuron loss [72, 73] and provide evidence that endogenous levels of mutant FUS protein are sufficient in animal models carrying identical gene dosage as in ALS-*FUS* patients.

The observed analogies between *Fus* ^*ΔNLS/*+^ mice and ALS-*FUS* indicate that *Fus* ^*ΔNLS/*+^ mice could be a model of choice to elucidate the cellular and molecular basis of ALS-*FUS*. In this respect, our study suggests that both cell-autonomous and non-cell autonomous toxicity contributes to trigger the motor phenotype in ALS-*FUS*. A previous study by Sharma and collaborators has demonstrated that motoneuronal expression of a FUS mutation is sufficient to trigger motor symptoms [72]. However, the expression of mutant FUS from the mostly neuronal TAU locus did not allow them to rigorously test the contribution of other FUS-expressing cell types. Here we show that loss of motor neuron cell bodies is completely rescued by reversal of the mutation in motor neurons. However, *Fus* ^*ΔNLS/*+^/*Chat*-CRE mice still developed motor symptoms after 10 months of age, and axonal damage appeared similar, independent of cytoplasmic FUS expression by motor neurons. In this knock-in *Chat*-CRE mouse strain, recombination occurs as early as E12 in motor neurons, and is extremely efficient [67]. Thus, both motor neuron autonomous and non-autonomous mechanisms contribute to motor neuron disease in *Fus* ^*ΔNLS/*+^ mice. These results are consistent with previous findings obtained in mutant SOD1 mice [8, 9, 93] as well as with results obtained in conditional transgenic TDP-43 mice (Da Cruz and Cleveland, personal communication). Hence, non-cell autonomous toxicity to motor neurons likely represents a ubiquitous mechanism of ALS.

Motor defects that are not rescued in *Fus* ^*ΔNLS/*+^/*Chat*-CRE mice could be due to defects in myelinating cells. Indeed, FUS was mislocalized in spinal cord oligodendrocytes of *Fus* ^*ΔNLS/*+^ mice and this mislocalization was maintained in *Fus* ^*ΔNLS/*+^/*Chat*-CRE mice. Consistent with a defect in oligodendrocytes, RNAseq uncovered decreased spinal cord expression of a number of genes that have been previously involved in morphology and function of myelin. Several of them are mutated in human demyelinating neuropathies, and loss of their function in mice leads to profound biochemical and morphological abnormalities of myelin. For instance, decreases in PMP2, whose mutations can cause demyelinating Charcot–Marie–Tooth disease (CMT) [57] is sufficient to modify the lipidome of peripheral myelin [94], while heterozygous loss of *Pmp22*, causing human peripheral neuropathy [60], disrupts myelin junctions in mice [28, 33]. Furthermore, loss of periaxin, causing CMT4F in humans [7, 27], is sufficient to profoundly alter the morphology of myelinating Schwann cells [15]. Importantly, we observed an increased number of oligodendrocytes in the ventral spinal cord white matter, consistent with altered function of this cell type, and this increased number was not dependent upon expression of the mutation in motor neurons as it persisted in *Fus* ^*ΔNLS/*+^/*Chat*-CRE mice. These results provide further evidence for the involvement of oligodendrocytes in ALS, and are consistent with results obtained in transgenic mice expressing mutant SOD1. In SOD1(G93A) mice, generation of oligodendrocytes from NG2+ cells is increased [40], and axons are abnormally myelinated in the grey matter [41, 63]. Interestingly, however, oligodendrocyte numbers appear increased in *Fus* ^*ΔNLS/*+^ mice, while they were reported to be unchanged in SOD1(G93A) mice. Such a difference might be due to the very different disease courses of both mouse strains. Oligodendrocytic expression of mutant SOD1 appears to play a critical role in mutant SOD1-ALS [41, 47, 63], and our current study provides suggestive, but not conclusive, evidence for a critical role of oligodendrocytes in ALS-*FUS*, and it does not identify pathogenic mechanisms elicited by mutant FUS in myelinating cells. Further work, using relevant CRE expressing lines and/or cell-specific gene expression profiling [76] is mandatory to explore this hypothesis.

Besides oligodendrocytes, we also provide evidence of defects in Schwann cells of *Fus* ^*ΔNLS/*+^ mice. There were myelin defects in ventral roots, and this was accompanied by increased CMAP latencies, characteristic for peripheral demyelination. Defects in Schwann cells have been recently observed in multiple models of ALS [37, 80], but expression of mutant SOD1 in Schwann cells appeared neither necessary [50] nor sufficient [83] for the disease triggered by this specific ALS-linked mutation, and the role of Schwann cells in ALS remains uncertain.

To our knowledge, myelin ultrastructure has not been systematically studied in ALS-*FUS* patients, yet several studies observed loss of myelin in the cortico-spinal tract of ALS-*FUS* patients [43, 84]. Moreover, FUS cytoplasmic aggregates have been observed in oligodendrocytes of ALS-*FUS* patients [51, 77] as well as in FTD patients with FUS pathology [58]. In non-*FUS* ALS patients, myelin loss has been observed in sporadic ALS patients and inclusions of TDP-43 are frequent in oligodendrocytes [41, 63]. Besides glial cells, other cell types, such as skeletal muscle, could contribute to the neuromuscular phenotypes, and the conditionality of *Fus* mutation in *Fus* ^*ΔNLS/*+^ mice will allow to investigate the role of these cells using appropriate CRE lines.

In conclusion, we characterize here a heterozygous knock-in mouse model of ALS and demonstrate that mutations in FUS result in a toxic gain of function leading to motor neuron disease through cell autonomous and non-cell autonomous mechanisms. *Fus* ^*ΔNLS/*+^ mice will be instrumental in deciphering the molecular derailments elicited by mutant FUS and could be useful for preclinical testing of therapeutic strategies.

## Electronic supplementary material

Below is the link to the electronic supplementary material.

Supplementary material 1 (PDF 2375 kb)
