# Supplementary material for: Motor neuron intrinsic and extrinsic mechanisms contribute to the pathogenesis of FUS-associated amyotrophic lateral sclerosis
Source: Acta Neuropathol. 2017 Feb 28;133(6):887–906. doi: 10.1007/s00401-017-1687-9 (PMC5427169; doi:10.1007/s00401-017-1687-9)
Supplement: Supplementary file 1 — Supplementary material 1 (PDF 2375 kb) [file 401_2017_1687_MOESM1_ESM.pdf]

Scekic-Zahirovic and collaborators

Motor neuron intrinsic and extrinsic mechanisms contribute to the pathogenesis of FUS-associated amyotrophic lateral sclerosis

Supplementary material

Supplementary Fig.1

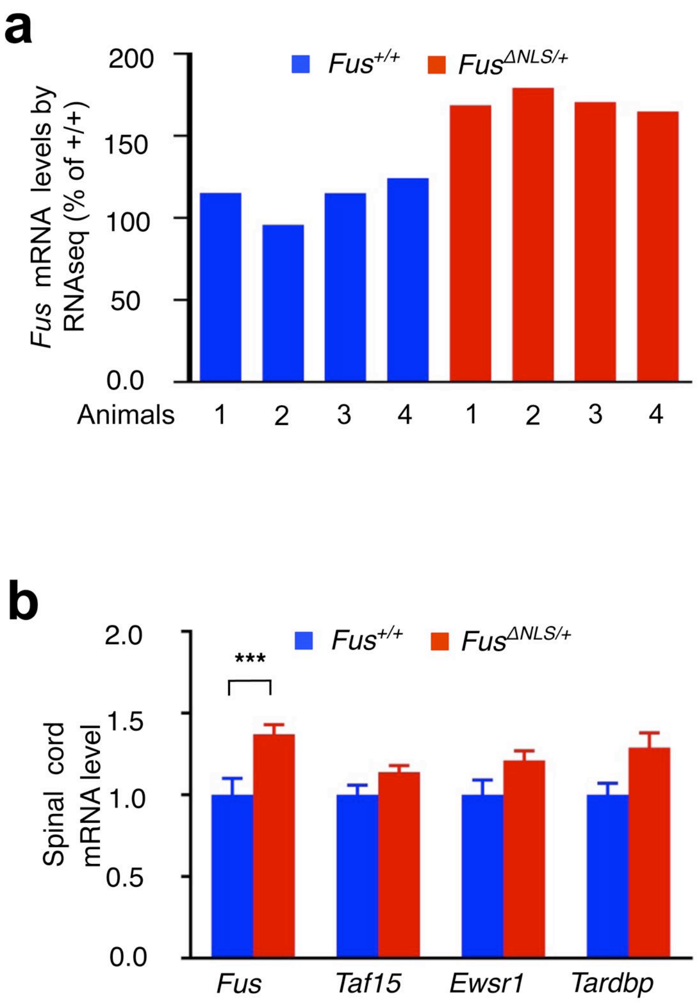

Supplementary Fig. 1: expression of *Fus* and related genes in *Fus*$^{\Delta NLS/+}$ spinal cord.

(a) Expression levels of total *Fus* (based on FPKM from RNA-seq) in spinal cord per animal.

(b) Expression levels of total *Fus*, *Taf15*, *Ewsr1* and *Tardbp* mRNA in spinal cord. *Fus* mRNA levels were significantly increased in tissues of *Fus*$^{\Delta NLS/+}$ knock-in mice as revealed by quantitative real-time PCR analysis. N=7 *Fus*$^{+/+}$, N=8 *Fus*$^{\Delta NLS/+}$. (***) $p<0.01$; Student's unpaired t-test.

Supplementary Fig. 2

a

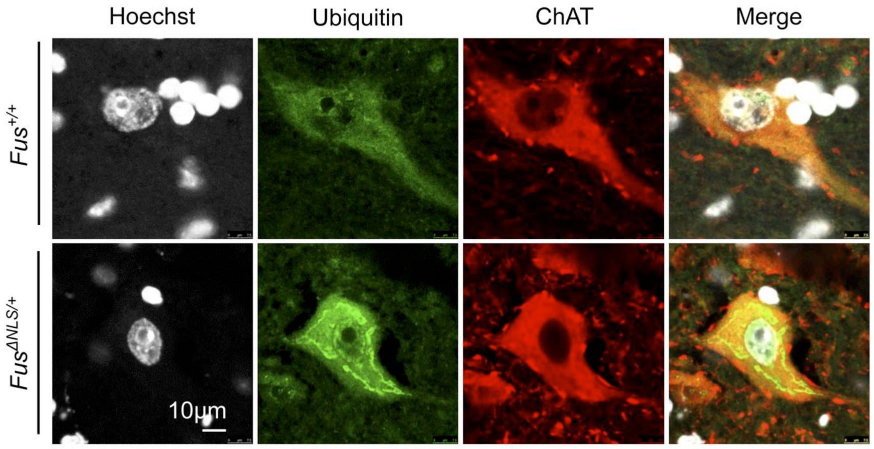

b

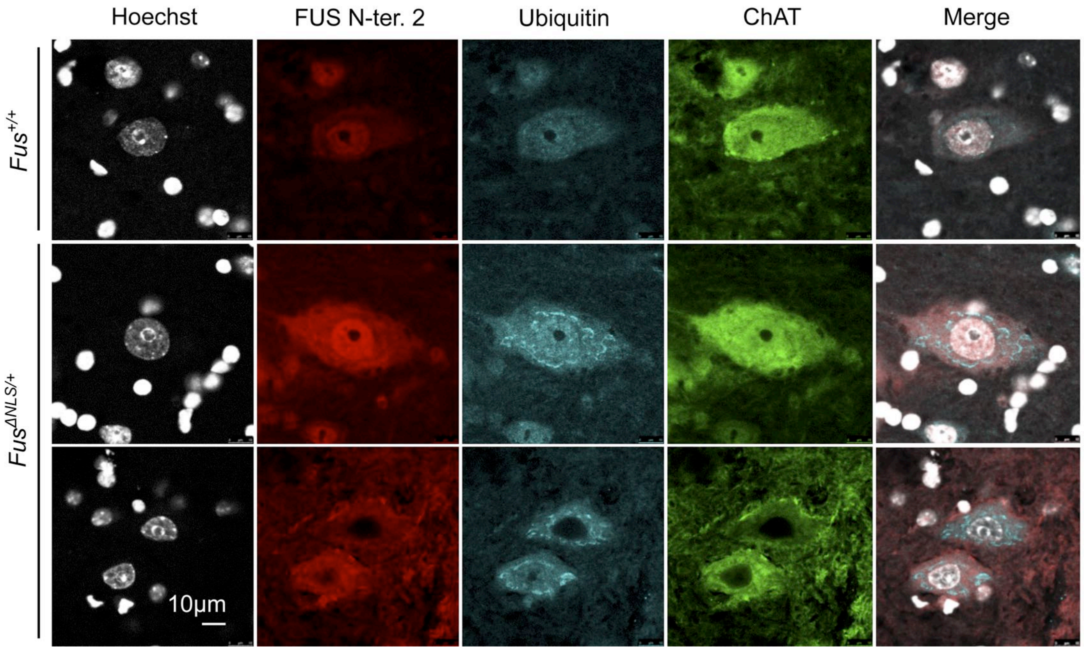

Supplementary Fig.2: K63-linked ubiquitin pathology in *Fus*$^{\Delta NLS/+}$ motor neurons.

(a) Ubiquitin-K63 linked immunofluorescence in a wild type *Fus*$^{+/+}$ (*upper panels*) and symptomatic *Fus*$^{\Delta NLS/+}$ (22 month-old) spinal cord (*lower panels*) showing ubiquitin pathology selectively in a population of large ChAT+ motor neurons (red). Ubiquitin (green) accumulation occured as large organized ubiquitin-positive cytoplasmic inclusions in *Fus*$^{\Delta NLS/+}$ motor neurons in contrast to diffuse cellular staining in *Fus*$^{+/+}$ motor neurons. Scale bars: 10 µm.

(b) Triple immunostaining with antibodies to the N terminus of FUS (red), ChAT (green) and Ubiquitin-K-63 linked (cyan). All neurons with clearance of nuclear FUS showed K-63 ubiquitin pathology. Hoechst staining is showing that nuclear structure is intact in neurons that have lost nuclear FUS staining. Notably, K-63 ubiquitin-positive aggregates were not enriched in FUS immunoreactivity. The ChAT signal was not included in the merge for clarity. Scale bars: 10 µm.

Supplementary Fig. 3

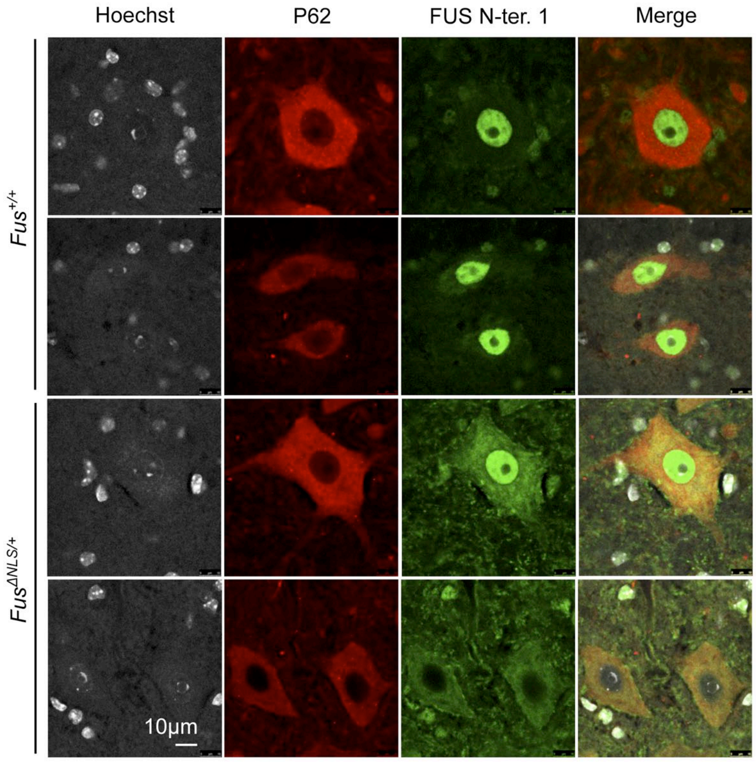

Supplementary Fig. 3: Lack of p62 pathology in *Fus*$^{\Delta NLS/+}$ motor neurons

Representative confocal images showing absence of p62 (red) pathology in *Fus*$^{\Delta NLS/+}$ (22 month-old) ventral horn cells co-labeled with FUS (green). Note that cells with exclusively cytoplasmic FUS also lack p62 positive aggregates.

Supplementary Fig. 4

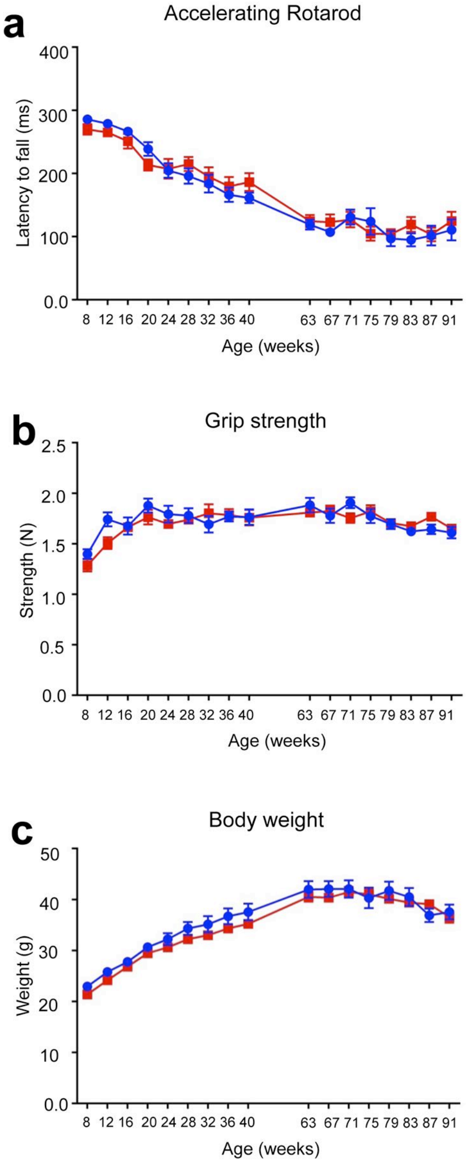

Supplementary Fig. 4 : Motor phenotype of *Fus*$^{\Delta NLS/+}$ mice.
(**a**) Accelerating rotarod **(b)** grip strength (**c**) and monthly body weight follow-up of 14 *Fus*$^{+/+}$ (blue) and 14 *Fus*$^{\Delta NLS/+}$ (red) male mice.

Supplementary Fig. 5

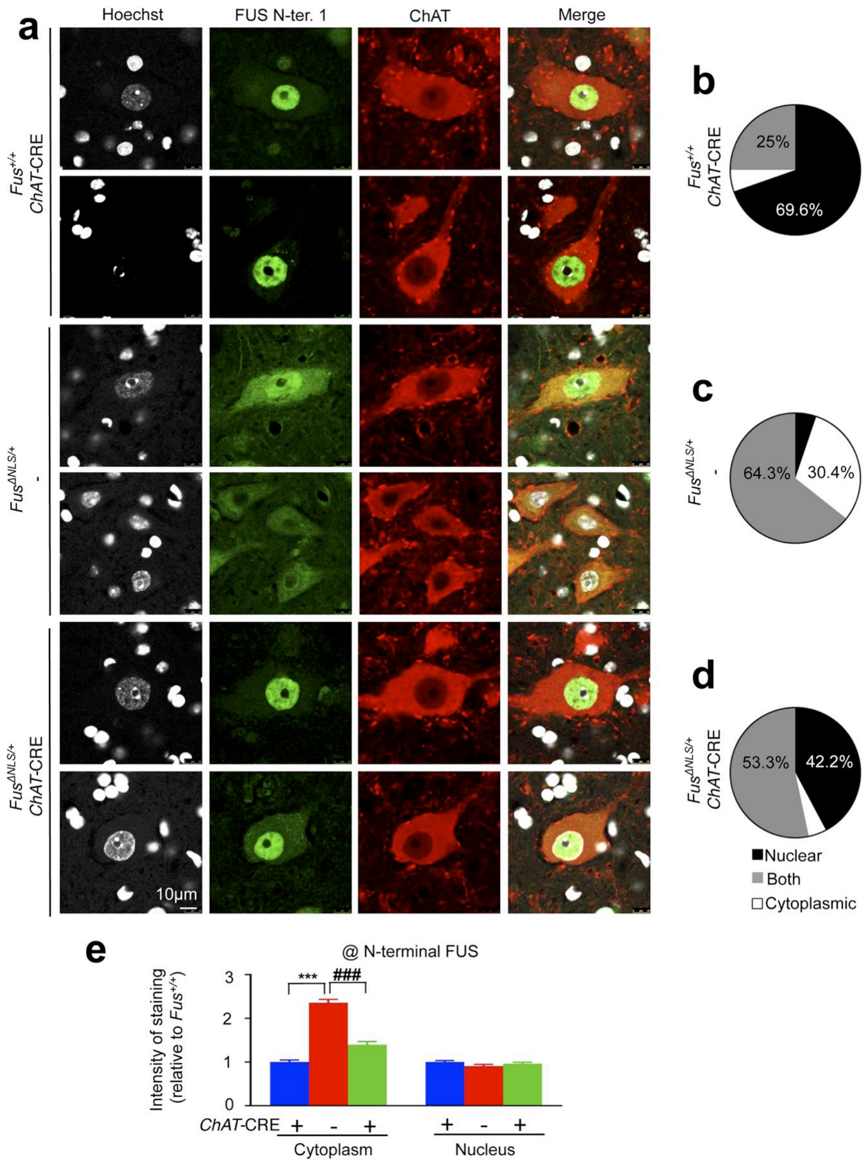

Supplementary Fig. 5: Restoration of FUS nuclear import in motor neurons

(a) Double-immunolabeling of spinal cord motor neurons with ChAT (red) and N-terminal FUS antibody (green). Nuclei were visualized with Hoechst (gray). Cellular localization of FUS was analyzed in the ventral spinal cord of *Fus*$^{+/+}$/*ChAT*-CRE, *Fus*$^{\Delta NLS/+}$/- , and *Fus*$^{\Delta NLS/+}$/*ChAT*-CRE mice.

(b-d) Analysis of FUS distribution within motor neurons showed that it was mainly nuclear in *Fus*$^{+/+}$/*ChAT*-CRE **(B)**, while enriched in the cytoplasm in *Fus*$^{\Delta NLS/+}$/- **(C).** In motor neurons of *Fus*$^{\Delta NLS/+}$/*ChAT*-CRE mice, FUS displayed either a nuclear or a mixed nuclear/cytoplasmic localization **(D)**. Thus, the ChAT-CRE allele induced successful recombination and partially restored FUS nuclear import in motor neurons.

(e) Quantification of FUS staining intensity (N-terminal antibody) in nucleus and cytoplasm of motoneurons confirmed reduced accumulation of FUS in the cytoplasm in *Fus*$^{\Delta NLS/+}$/*ChAT*-CRE compared to *Fus*$^{\Delta NLS/+}$/-. N=112 *Fus*$^{+/+}$/*ChAT*-CRE (blue), N=86 *Fus*$^{\Delta NLS/+}$/- (red). N=89 *Fus*$^{\Delta NLS/+}$/*ChAT*-CRE (green). (***) p<0.01 versus *Fus*$^{+/+}$/*ChAT*-CRE, (###) p<0.01 versus *Fus*$^{\Delta NLS/+}$/*ChAT*-CRE. One way ANOVA followed by Tukey *post hoc* test.

Supplementary Fig. 6

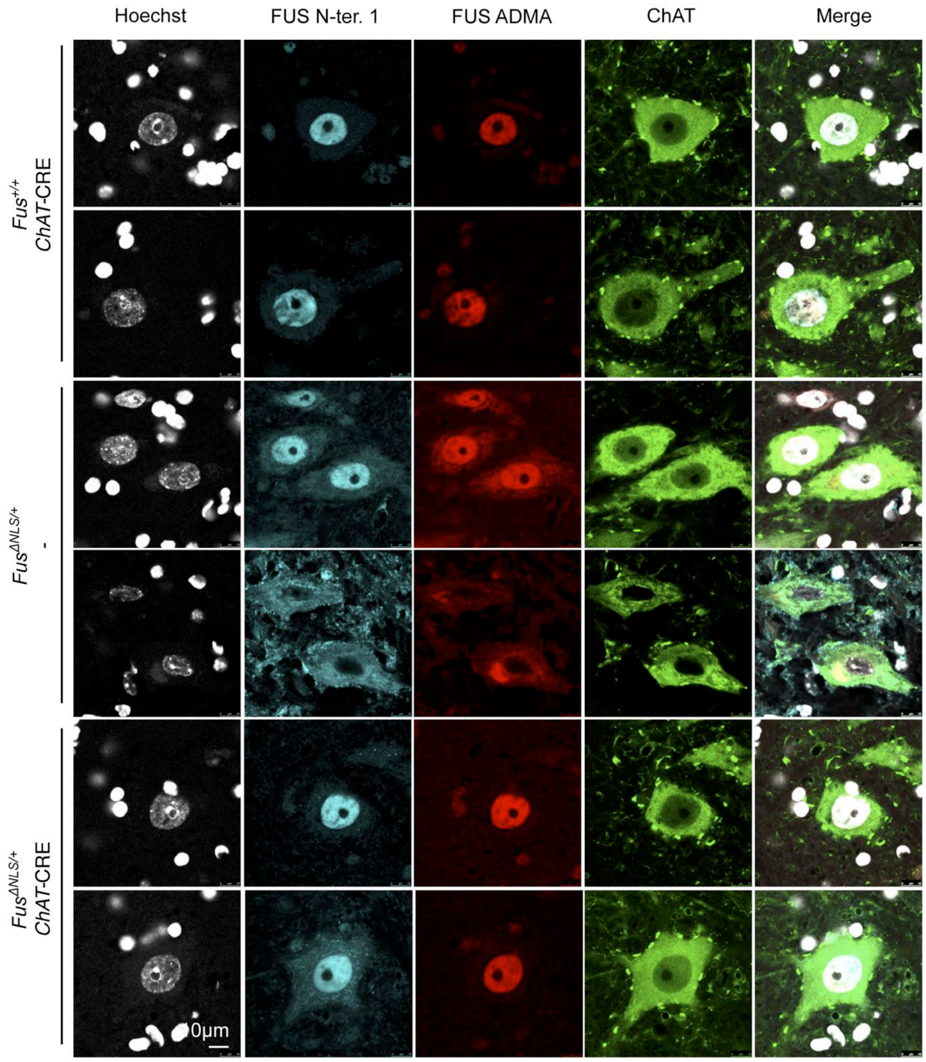

Supplementary Fig. 6: Rescue of ADMA-FUS nuclear import in motor neurons upon CRE recombination
Triple immunolabeling for the ChAT (green), FUS (N-terminal antibody) (cyan) and ADMA-FUS (red) in the spinal cord ventral horn. ADMA-FUS nuclear localization was restored in motor neurons of *Fus*$^{\Delta NLS/+}$/*ChAT*-CRE mice.

Supplementary Fig. 7

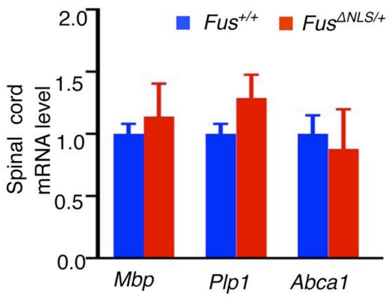

Supplementary Fig. 7: Expression of additional myelin related genes in *Fus*$^{\Delta NLS/+}$ spinal cord.
mRNA expression levels of myelin-related genes in spinal cord at 22 months of age. N=7. No significant difference observed; Student's unpaired t-test.

Supplementary Fig. 8

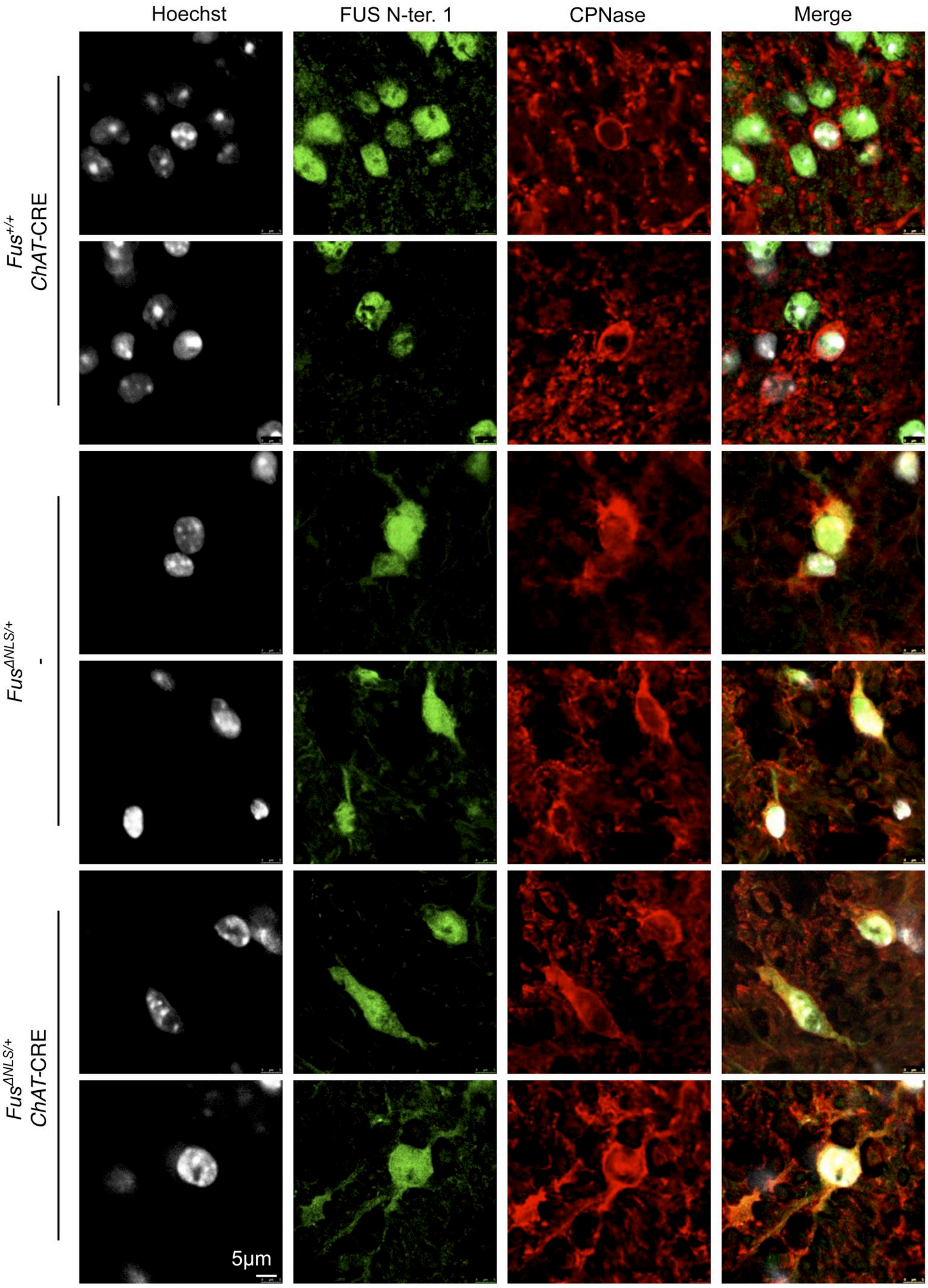

Supplementary Fig.8: cytoplasmic FUS mislocalization in *Fus*$^{\Delta NLS/+}$ oligodendrocytes

Double immunostaining for FUS (N-terminal part) (green) and the oligodendrocyte marker CNPase (red). Note presence of intense cytoplasmic FUS staining in both *Fus*$^{\Delta NLS/+}$/- and *Fus*$^{\Delta NLS/+}$/*ChAT*-CRE compared with exclusive nuclear FUS staining in *Fus*$^{+/+}$/*ChAT*-CRE oligodendrocytes.

Supplementary Fig. 9

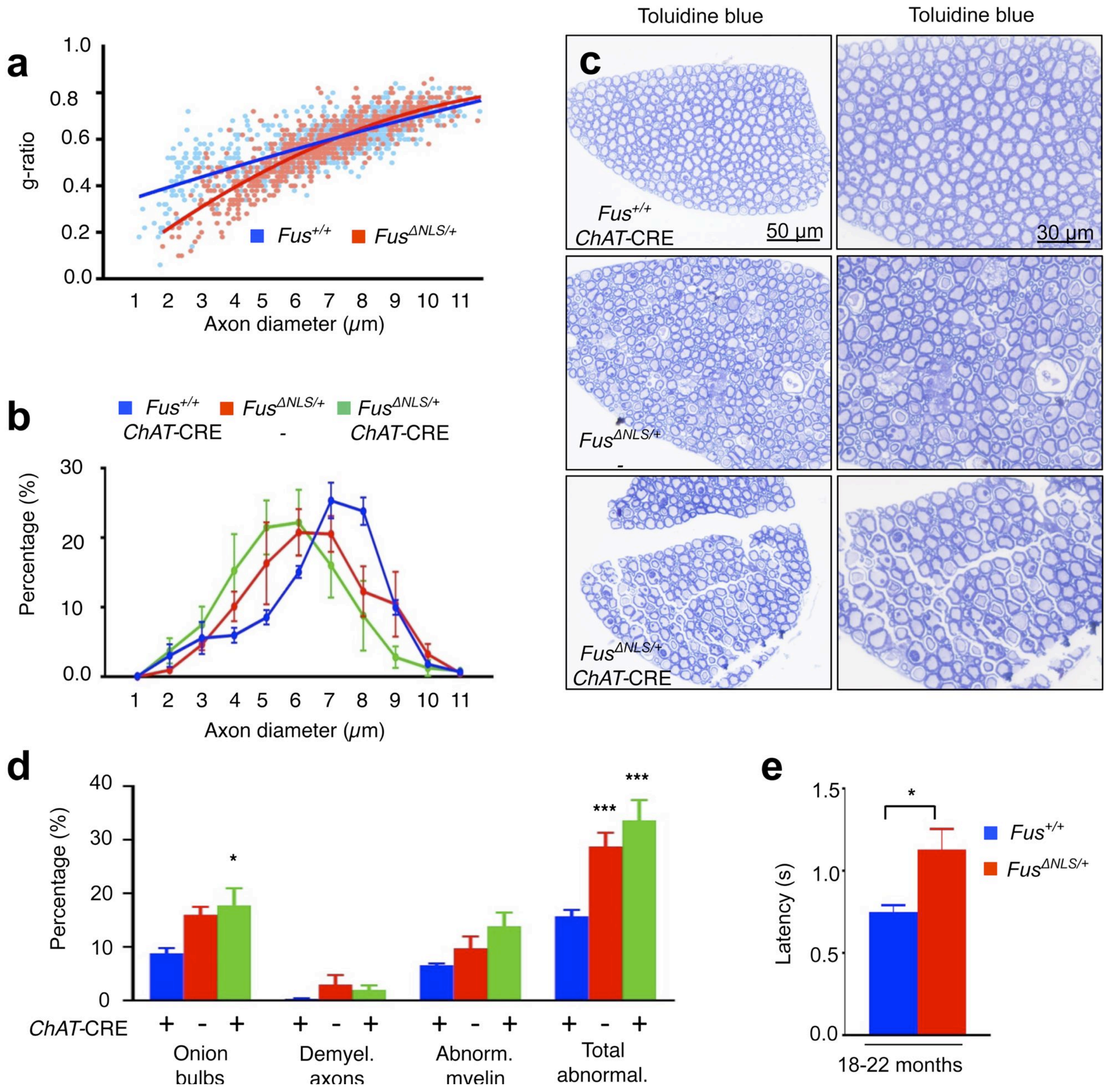

Supplementary Fig.9: Axonal and myelin abnormalities in *Fus*$^{\Delta NLS/+}$ mice

(a) G-ratio of ventral root axons as a function of axonal diameter in *Fus*$^{\Delta NLS/+}$ (red dots) compared to their control littermates (blue dots). A best fit polynomial regression line is drawn for each genotype (*Fus*$^{\Delta NLS/+}$ in red and *Fus*$^{+/+}$ in blue)

(b) Distribution of axonal diameter in ventral roots of *Fus*$^{\Delta NLS/+}$/- (red), *Fus*$^{\Delta NLS/+}$/*ChAT*-CRE (green) and *Fus*$^{+/+}$/*ChAT*-CRE (blue) mice.

(c) Representative images of toluidine blue stained L4 ventral nerve roots. Note aberrant myelin features throughout the transverse nerve root section in both *Fus*$^{\Delta NLS/+}$ genotypes. Scale bar: 50 µm for lower and 30µm for higher magnification.

(d) Myelin abnormalities in *Fus*$^{\Delta NLS/+}$/- (red), *Fus*$^{\Delta NLS/+}$/*ChAT*-CRE (green) and *Fus*$^{+/+}$/*ChAT*-CRE (blue) mice. The following subcategories of myelin abnormalities are shown : onion bulbs (as a sign of demyelination and remyelination), demyelinated axons, abnormal myelin foldings. The last series of bars sums up all myelin abnormalities per genotype. (*) $p<0.05$, (***) $p<0.01$ versus *Fus*$^{+/+}$/*ChAT*-CRE; One way ANOVA followed by Tukey *post hoc* test.

(e) Bar graphs showing means and standard errors for compound muscle action potential (CMAP) latency time. (*) $p<0.05$ as compared to *Fus*$^{+/+}$; N=10 *Fus*$^{+/+}$, N=9 *Fus*$^{\Delta NLS/+}$. Student's unpaired t-test.

Supplementary Table 1: oligonucleotides used for RT-qPCR

Gene	**Forward primer**	**Reverse primer**
Abca1	CGACCATGAAAGTGACACGC	AGCACATAGGTCAGCTCGTG
Cldn19	GGAGAGGGCGAACAGCATC	AACTTAACAACAGGTTCTCTGGC
Dhh	AGCAACTTGTGCCTCTGCTA	TAGTTGGGTACGAGGTCCCG
Ewsr1	GAGGAAATGTCCAGCACCGA	GGCTTAGGGGCCTTACACTG
Fus	TTATGGACAGACCCAAAAACACA	TGCTGCCCATAAGAAGATTG
Mbp	CAGAAGAGACCCTCACAGCG	CTCTGTGCCTTGGGAGGAAG
Myocillin	TGGAATTTGGACACGTTGGC	CCTTCTTTGCTCCTGGGTCG
NcMap	AACATGACCAGGGGAGAGGA	TCAGCAGGATCAGCACCAAG
Plp1	TGTGGCTCCAACCTTCTGTC	GCGAAGTTGTAAGTGGCAGC
Pmp2	GGCACCTGGAAGCTTGTCTC	CTGTTGGCTAACCCCACACC
Pmp22	CTGGTCTGTGCGTGATGAGT	CAGGATGTAGGCGAAGCCAT
Prx	AGTGGCCAAGCTGAACATCC	AGAACTCGACGTCAACAGGG
Taf-15	CGGCTGTTAGTCATGTCGGA	TGTCCATAGCCTTGACTGCC
Tardbp	TCCAGGTGGCTTTGGGAATC	CAAAGTTCATCCCTCCACCCA
